# Stepwise combined cell transplantation using mesenchymal stem cells and induced pluripotent stem cell-derived motor neuron progenitor cells in spinal cord injury

**DOI:** 10.1186/s13287-024-03714-3

**Published:** 2024-04-23

**Authors:** Jang-Woon Kim, Juryun Kim, Hyunkyung Mo, Heeju Han, Yeri Alice Rim, Ji Hyeon Ju

**Affiliations:** 1 CiSTEM laboratory, Catholic iPSC Research Center (CiRC), College of Medicine, The Catholic University of Korea, 06591 Seoul, Republic of Korea; 2 Department of Biomedicine & Health Science, Seoul St. Mary’s Hospital, College of Medicine, The Catholic University of Korea, 06591 Seoul, Republic of Korea; 3 YiPSCELL, Inc, Seoul, South Korea; 4 Division of Rheumatology, Department of Internal Medicine, Seoul St. Mary’s Hospital, Institute of Medical Science, College of Medicine, The Catholic University of Korea, 06591 Seoul, Republic of Korea

**Keywords:** Mesenchymal stem cells, Induced pluripotent stem cells, Motor neuron progenitor cells, Spinal cord injury, Cell transplantation

## Abstract

**Background:**

Spinal cord injury (SCI) is an intractable neurological disease in which functions cannot be permanently restored due to nerve damage. Stem cell therapy is a promising strategy for neuroregeneration after SCI. However, experimental evidence of its therapeutic effect in SCI is lacking. This study aimed to investigate the efficacy of transplanted cells using stepwise combined cell therapy with human mesenchymal stem cells (hMSC) and induced pluripotent stem cell (iPSC)-derived motor neuron progenitor cells (iMNP) in a rat model of SCI.

**Methods:**

A contusive SCI model was developed in Sprague-Dawley rats using multicenter animal spinal cord injury study (MASCIS) impactor. Three protocols were designed and conducted as follows: (Subtopic 1) chronic SCI + iMNP, (Subtopic 2) acute SCI + multiple hMSC injections, and (Main topic) chronic SCI + stepwise combined cell therapy using multiple preemptive hMSC and iMNP. Neurite outgrowth was induced by coculturing hMSC and iPSC-derived motor neuron (iMN) on both two-dimensional (2D) and three-dimensional (3D) spheroid platforms during mature iMN differentiation in vitro.

**Results:**

Stepwise combined cell therapy promoted mature motor neuron differentiation and axonal regeneration at the lesional site. In addition, stepwise combined cell therapy improved behavioral recovery and was more effective than single cell therapy alone. In vitro results showed that hMSC and iMN act synergistically and play a critical role in the induction of neurite outgrowth during iMN differentiation and maturation.

**Conclusions:**

Our findings show that stepwise combined cell therapy can induce alterations in the microenvironment for effective cell therapy in SCI. The in vitro results suggest that co-culturing hMSC and iMN can synergistically promote induction of MN neurite outgrowth.

**Graphical Abstract:**

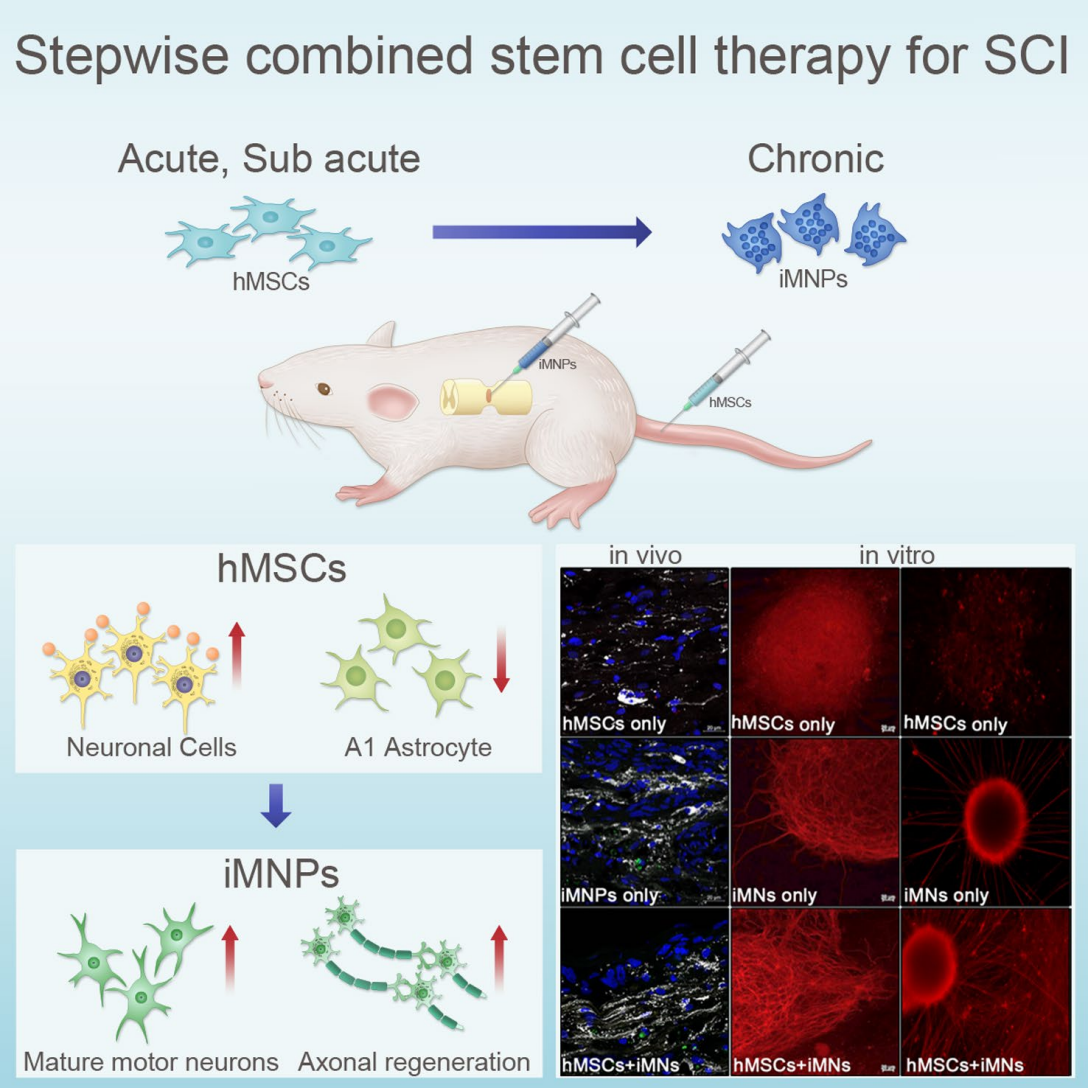

**Supplementary Information:**

The online version contains supplementary material available at 10.1186/s13287-024-03714-3.

## Background

Contusive spinal cord injury (SCI) can induce permanent impairment of the sensory and motor nervous systems, leading to restricted muscle movement. Moreover, patients with SCI have a lower quality of life in comparison to their uninjured peers due to low life expectancy and lower employment rates [1, 2]. The pathophysiologic mechanisms of SCI may be primary or secondary phase injury. Primary SCI is caused by direct traumatic injury to the spinal cord (SC). On the other hand, secondary SCI can be categorized into acute, subacute, and chronic phases according to the time and pathological mechanisms after the primary injury [3]. Secondary injury mechanisms include neuronal and glial cell death, primarily due to apoptosis and autophagy in the injured regions. In addition, secondary injuries activate astrocytes over time, resulting in reactive gliosis and subsequent glial scar formation, which acts as a physical and chemical barrier inhibiting axonal regeneration [4, 5]. Therefore, the goal of SCI treatment in clinical practice is to minimize secondary injuries after the primary injury. Therapeutic attempts to overcome SCI are generally based on two pathophysiological mechanisms: neuroprotective and neuroregenerative approaches [3, 5, 6]. 

Stem cell therapy has demonstrated both neuroprotective and neuroregenerative effects in SCI by minimizing the pathophysiological mechanisms that occur during primary and secondary injuries [7–9]. The neuroprotective mechanisms of the transplanted cells include secondary injury mitigation by protecting the injured region of the SC. The basic mechanism has been reported to be preservation of the adjacent tissues at the injured site by stem cells in comparison to the control group [5, 10]. Remyelination after SCI is a therapeutic target in regenerative trials. Myelination plays a critical role in effective action potential propagation along with survival of axons and corresponding neurons in the SC tissue. Enhanced approaches for cell transplantation and endogenous repair processes are being actively investigated to improve remyelination in SCI [3]. Numerous cell types have been transplanted and studied for their neuroprotective and neuro-regenerative effects in SCI. Several cell types, including Schwann cells, neural stem and progenitor cells (NSPCs), mesenchymal stem cells (MSCs), olfactory ensheathing cells (OECs), and oligodendrocyte precursor cells, are being studied in the context of promoting neuroprotection in SCI [10–12]. The therapeutic effects of different types of differentiated cells derived from multipotent or pluripotent cell types are also being investigated in SCI [5, 13]. Till date, various cell types have been successfully differentiated from induced pluripotent stem cells (iPSCs) and transplanted into animal models of SCI, suggesting their potential for neuroprotection and neuroregeneration [5, 14]. Moreover, several studies have reported the efficacy of differentiated cell types derived from iPSCs in preclinical and clinical SCI trials [15–17]. 

Unfortunately, the ideal cell type for complete repair and regeneration of the SC has not yet been determined [15]. Several clinical trials of stem cell transplantation for SCI have been conducted; however, no alternatives to the most effective methods have been found and there is no consensus regarding transplantation timing, cell types, cell capacity, and transplantation route [17]. For effective stem cell therapy in SCI, trials using different cell types and transplantation strategies for each phase of SCI are warranted [5, 18, 19]. 

Previous studies on embryonic stem cell (ESC)-derived motor neuron progenitor cells (MNPs) transplanted in the SC of adult rats after SCI have shown enhanced endogenous axon sprouting and functional improvement [5, 20–22]. Stem cell-derived motor neurons (MNs) are increasingly being used as a cell replacement strategy for neuroregeneration in SCI. Recently, several protocols have been established to generate MNPs and MNs from iPSCs. However, the cellular and molecular mechanisms of iPSC-derived MNP (iMNP) in different phases of SCI require further investigation.

In this study, we confirmed iMNP engraftment and MN differentiation at the injury site in a rat model of chronic SCI (Subtopic 1). However, the transplanted iMNPs did not decrease the expression of reactive gliosis at the injury site. MSC transplantation in SCI induces the secretion of various neurotrophic factors and cytokines at the lesional site, and has immunomodulatory, anti-apoptotic, and anti-inflammatory effects [23, 24]. Based on the findings of the aforementioned studies, we attempted to apply both MSC and iMNP to increase the efficacy of cell therapy at the lesional site.

We performed stepwise combined cell transplantation using MSC and iMNP in a severed chronic SCI model (Main topic). We hypothesized that preemptive multiple MSC transplantation during the acute phase (Subtopic 2) might induce microenvironmental alterations at the lesional site, and subsequent iMNP transplantation during the chronic phase would enhance the differentiation of transplanted cells into mature MN and induce axonal regeneration along with functional recovery. We demonstrate that stepwise combined cell transplantation using MSCs and iMNP in SCI promotes mature MN differentiation and axonal regeneration at the injury site. Moreover, stepwise combined cell transplantation significantly improved behavioral recovery in rat models of SCI compared to single-cell therapy. The in vitro results confirmed that MSC and iMN act synergistically and play a critical role in the induction of neurite outgrowth during MN differentiation and maturation.

## Materials and methods

### Animals and contusive SCI model

All the surgical and cell transplantation procedures in animals were reviewed and approved by the Animal Studies Committee of the School of Medicine, the Catholic University of Korea (IACUC approval Number CUMC-2020-0044-04). All surgical procedures, including pre- and post-cell transplantation procedures, were performed in accordance with the Laboratory Animal Welfare Act, the Guidelines and Policies for Rodent Survival Surgery, and ARRIVE (Animal Research: Reporting of In Vivo Experiments). Euthanasia was performed using CO_2_ gas for all the animals in accordance with the American Veterinary Medical Association (AVMA) Guidelines for Euthanasia of Animals, 2020 edition. A contusive SCI model was prepared as previously described [8, 9]. In brief, adult male Sprague–Dawley (SD) rats (body weight, 280–320 g) were anesthetized with isoflurane via inhalation and Rompun (2 mg/kg) via intraperitoneal injection. The backs of all rats were shaved, disinfected with ethanol, and sterilized using antiseptic betadine. After exposing the paravertebral muscles from T8-T10, laminectomy was performed at T9 level. Contusion SCI was induced using the Multicenter Animal Spinal Cord Injury Study (MASCIS) impactor (a rod weighing 10 g was dropped from a height of 2.5 cm). Pre- and postoperatively, 5 mg ketoprofen and gentamicin were administered for five days. After surgery, 3 mL warm saline solution was injected subcutaneously. The bladder was manually emptied for one 1 week.

### Subtopic 1; chronic SCI + iMNP transplantation

#### Generation of iMNP

Human iPSCs were generated from peripheral blood mononuclear cells (PBMCs) using the CytoTune-iPS Sendai Reprogramming Kit containing Yamanaka factors (A16518, Thermo Fisher Scientific, Waltham, MA, USA), as described previously [25–28]. These iPSCs were cultured and maintained in Essential 8 medium (E8, Thermo Fisher Scientific) in vitronectin-coated dishes. Human iPSC-derived MNPs and MNs were generated according to a previously reported protocol [19]. Human iPSCs (1.3 × 10^5^/ 6-well plate) were cultured in laminin-coated petri dishes. The following day, iPSC medium was replaced with a chemically defined neural induction medium, including Dulbecco’s Modified Eagle’s Medium (DMEM)/F12, neurobasal medium at a ratio of 1:1, 1% N2, 1% B27 (Thermo Fisher Scientific), 0.1 mM ascorbic acid (Sigma-Aldrich, St Louis, MO, USA), 1X Glutamax, and 1X penicillin/streptomycin (Thermo Fisher Scientific). CHIR99021 (3 µM, Tocris, Bristol, United Kingdom), 2 µM dorsomorphin homologue 1 (DMH1, Tocris), and 2 µM SB431542 (Stemgent, Cambridge, MA, USA) were added to the neural induction medium to induce iNEP differentiation. The culture medium was changed consecutively for six days. The differentiated iNEP cells were dissociated using Accutase (Innovation Cell Technology, Inc., CA, USA) and 1.3 × 10^6^ / six-well plate in laminin-coated plate dishes. Subsequently, RA (0.1 µM, Stemgent) and Pur (0.5 µM, Stemgent) were added to the iNEP cells in combination with 1 µM CHIR99021 (Torcris), 2 µM DMH1 (Tocris), and 2 µM SB431542 (Tocris) for six days to differentiate into iMNP. Subsequently, iMNP was dissociated using Accutase and cultured in a suspension of neural induction medium with 0.5 µM RA and 0,1 µM Pur to induce the differentiation of iMN for additional six days. For maturation, iMN was cultured with 0.5 µM RA, 0.1 µM Pur, and 0.1 µM Compound E (Calbiochem, San Diego, CA, USA) for 10 days.

#### In vivo cell transplantation and stepwise cell therapy

For the subtopic 1 concept, rats were randomized into the following groups at six weeks post-SCI for transplantation: (1) SCI + PBS group (*n* = 4), (2) SCI + iMNP (12 weeks) group (*n* = 3), and (3) SCI + iMNP (14 weeks) group (*n* = 2). IL transplantation of 1 × 10^6^ iMNP cells in the injured SC was performed. The injured site was re-exposed, and 1 × 10^6^ iMNP (PKH26 labeling) cells in 10 µL PBS was transplanted using a Hamilton needle.

### Subtopic 2; Acute SCI + multiple hMSC transplantation

#### hMSC culture

Bone marrow-derived hMSCs were purchased from the Catholic Institute of Cell Therapy, South Korea. The purchased hMSCs were cultured in DMEM (Thermo Fisher Scientific) supplemented with 10% fetal bovine serum, 100 U/mL penicillin, and 100 µg/mL streptomycin (Thermo Fisher Scientific) in a 5% CO_2_ humidified atmosphere at 37 ℃. Passage 5 MSCs were used for transplantation.

#### In vivo cell transplantation and stepwise cell therapy

For the subtopic 2 concept, rats were randomly assigned to one of the following three groups before MSC transplantation at 24 h post-SCI: (1) SCI + PBS group (*n* = 7), (2) SCI + 1’MSCs group (*n* = 5), and (3) SCI + 2’MSCs group (*n* = 6). MSC transplantation with 1 × 10^6^ MSCs (PKH26 labeling) was performed in each group 24 h and 1-week post injury. For IV injections, 1 × 10^6^ cells in a total volume of 100 µL PBS were injected through the tail vein using an insulin syringe.

### Main topic; chronic SCI + stepwise combined cell therapy using preemptive multiple hMSC and iMNP

#### In vivo cell transplantation and stepwise cell therapy

For the main topic concept, rats were randomly assigned to one of the following four groups before preemptive MSC transplantation at 24 h post-SCI: (1) SCI + PBS group (*n* = 10), (2) SCI + 2’MSC group (*n* = 10), (3) SCI + iMNP group (*n* = 8), and (4) SCI + 2’MSCs + iMNP group (*n* = 10). Based on the results of previous concepts, we performed stepwise cell therapy by injecting iMNP six-weeks after MSC transplantation. Preemptive cell therapy was performed with 1 × 10^6^ MSCs (PKH26) via IV injection at 24 h and one-week post injury. At six-weeks post injury, the lesional and injured sites were re-exposed, and 1 × 10^6^ iMNPs (PKH67) were transplanted. Intramuscular injections of cyclosporin A (Cipol Inj, Chongkundang Pharmaceutical) at a dosage of 10 mg/kg were administered daily starting the day after iMNP transplantation. The sample size calculation of the animal model was performed based on the previously reported efficacy of neuroregeneration using hMSC and iMNP transplantation in SCI model [7, 8, 21, 22, 29–31]. 

#### BBB locomotor rating scale

After SCI, the clinical behavioral outcomes were assessed using the BBB locomotor rating scale. BBB locomotor rating scale was also used before transplantation; and rats with significant spontaneous improvement in hind limb function were excluded from the experiment. The BBB locomotor rating scale scores were recorded preoperatively, pre-transplantation, and every week by two independent researchers after a 2-min observation period. Based on the BBB locomotor rating scale scores, the rats were divided into three grades: grade 1, scores 0–5; grade 2, scores 5–10; and grade 3, scores 10–15. Comparative analysis of incidence rates was performed for grade 3.

Incidence rates (%) were calculated as follows:

(BBB grade score total rats / total rats) × 100.

#### Cell labeling for in vivo tracking

Before cell transplantation, the cells were labeled with fluorescent membrane-intercalating dyes PKH26 (red fluorescence) and PKH67 (green fluorescence; Sigma-Aldrich). Cells were collected, washed once using serum-free medium, and centrifuged at 2,500 rpm for 5 min. After aspirating the supernatant, the cell pellet was incubated with Diluent C, and PKH26 and PKH67 dye solutions for 5 min at room temperature (RT). Cell labeling with PKH26 and PKH67 dyes was terminated using 1% bovine serum albumin (BSA). After the final wash, cells were centrifuged and the cell pellet was suspended in PBS and used for cell transplantation.

#### MEA analysis

MEA was used to confirm the electrophysiological parameters of the cells that differentiated towards the MN lineage. Before cell seeding, 10 µL of 0.1% polyethyleneimine solution was incubated over the electrode array of each well in the MEA plate for 1 h at RT. Afterwards, the MEA plate was rinsed with sterile deionized water thrice, and water was aspirated from the MEA plate. The samples were then dried overnight in a biosafety cabinet at RT. Subsequently, 1 × 10^6^ cells treated with laminin (10 µg/mL) were seeded onto the MEA plate. After 1 h, the cells were incubated with neural induction medium. Once the cells were in place, the MEA plate was incubated in a cell culture incubator in a 5% CO_2_ humidified atmosphere at 37 ℃ for 3–4 h. Neuronal electroactivity was monitored the next day and the spike number for differentiated iPSC-derived MN was recorded.

#### Antibodies

MN differentiation was evaluated in vitro and in vivo using SOX1, OLIG2, ChAT, HB9, and SMI-32 antibodies. Cell proliferation was evaluated using Ki-67 antibody. Engraftment of the transplanted hMSCs was performed using GFAP (astrocyte), C3 (A1 astrocyte type), CC-1 (oligodendrocyte), and NeuN (neuron) antibodies. Neurocan was used to assess the extracellular matrix molecules at the lesional site. Expression of growth factors was evaluated based on the expression of NGF, BDNF, and VEGF. Synapse formation and axonal sprouting were evaluated based on the expression of synapsin-1 and MAP-2 at the lesional site. Details of the primary antibodies used are listed in Table 1.

**Table 1:** List of Antibodies, Host, and Dilutions information

Primary Antibody	Host	Dilution	Manufacturer
**Motor neuron differentiation**			
SOX1	Goat	1:50 †	R&D SYSTEMS
OLIG2	Rabbit	1:50 †	Millipore
		1:700 *	
ChAT	Goat	1:50 †	Millipore
		1:700 *	
HB9	Rabbit	1:50 †	Millipore
SMI-32	Mouse	1:50 †	BioLegend
		1:700 *	
**Cell proliferation**			
Ki-67	Goat	1:50 †	Santa cruz
**Neuron, Astrocyte, Reactive Astrocyte and Oligodendrocyte**			
Neu N (Neuron)	Mouse	1:100 †	Abcam
		1:700 *	
GFAP (Astrocyte)	Mouse	1:100 †	Merck
		1:700 *	
	Rabbit	1:1000 †	Abcam
		1:10000 *	
C3 (Reactive astrocyte, A1)	Rabbit	1:500 †	Abcam
		1:1000 *	
CC-1 (Oligodendrocyte)	Mouse	1:100 †	Merck
		1:700 *	
**Growth factors**			
NGF	Rabbit	1:50 †	Abcam
		1:700 *	
BDNF	Rabbit	1:50 †	GeneTex
		1:700 *	
VEGF	Mouse	1:50 †	Santa cruz
		1:700 *	
**Synaptogenesis and Axonal regeneration**			
Synapsin-1	Rabbit	1:50 †	Sigma-Aldrich
		1:700 *	
MAP-2	Mouse	1:50 †	Santa cruz
		1:700 *	
**House Keeping Protein**			
GAPDH	Mouse	1:2000 *	Abcam
Beta actin	Rabbit	1:2000 *	Abcam

* Dilution of immunofluorescence staining (Dilution:)

† Dilution of western blot

#### Cell proliferation assay

Proliferation assays were performed using the CCK-8 assay (Sigma-Aldrich) to confirm iPSC-derived iMNP, iMN, and iMature MN proliferation at each stage of MN differentiation. After seeding 5 × 10^4^ cells in laminin-coated plate wells, the cells were incubated in 100 µL of media in a 5% carbon dioxide (CO_2_) humidified atmosphere at 37 ℃. Subsequently, 10 µL of the CCK-8 solution was added to each well of the plate. After incubating the plate for 4 h, absorbance was measured at 450 nm using a microplate reader. At each stage of differentiation of iNEPs, iMNPs, and MNs, cell proliferation was measured at 2, 4, and 6 days post cell seeding.

#### Immunofluorescence staining of in vitro cultured cells

For in vitro differentiation, cells were seeded onto laminin-coated coverslips. For iNEP staining, 5.8 × 10^4^ cells were seeded, and 5 × 10^5^ cells were seeded to stain iMNP, iMN, and iMature MNs. After six days of cell culture, in the iNEP, iMNP, and iMN stages, immunofluorescence staining was performed. For the iMature MN stage, immunofluorescence staining was performed after 10 days of culture. The differentiated cells were fixed in 4% paraformaldehyde (PFA) for 30 min at RT. The cells were permeabilized with 0.1% Triton X-100 for 10 min at RT. Cells were blocked with PBS containing 2% BSA (PBA, Sigma-Aldrich) for 30 min at RT. Primary antibodies were diluted and incubated with 2% PBA for 2 h at RT. After washing, Alex Fluor-488 or -594 (Life Technologies) conjugated secondary antibodies diluted in 2% PBA were added and incubated at RT for 1 h. Cells were stained with 4′,6-diamidino-2-phenylindole (DAPI, Roche, Basel, Switzerland), washed, and mounted using an antifade mounting reagent (Thermo Fisher Scientific). The cells were observed under a fluorescence microscope (Carl Zeiss, Oberkochen, Germany) (x 200 magnification).

#### Immunofluorescence staining using SC samples

At 6- and 12-weeks post injury, euthanasia was performed using compressed CO_2_ gas, which was injected at a rate of 30 $\sim$ 70% of the chamber volume/min. Cardiac arrest was confirmed after euthanasia, and tissue sampling was performed. The injured SCs were obtained after transcardial perfusion with PBS and 4% PFA, fixed overnight in 4% PFA, and then incubated overnight in 15% and 30% sucrose solutions. Subsequently, the tissue samples were embedded in optimal cutting temperature (OCT) embedding matrix compound (Tissu-Tek; Sakura Finetek USA, Torrance, CA, USA) and snap-frozen in liquid nitrogen. Injured SC Sect. (4-µm thickness) were obtained and mounted on saline-coated slides. The SC sections were permeabilized with 0.1% Triton X-100 for 20 min at RT. Sections were blocked with normal goat or horse serum containing 0.1% Triton X-100 for 1 h at RT. Primary antibodies were diluted and incubated with a mixture of 0.1% Triton X-100 overnight at 4 ℃. After washing, Alex Fluor-488 or -594 and Cy5 (Life Technologies, Carlsbad, CA, USA)-conjugated secondary antibodies were diluted in Tris-buffered saline (TBS) containing 0.05% Tween-20 (TBST) at RT for 1 h. Slides were stained with DAPI, finally washed, and mounted using an antifade mounting reagent. Stained tissues were observed using a confocal microscope, LSM900 (Carl Zeiss, Oberkochen, Germany).

#### Western blot analysis

Lesion segments ($\sim$ 1 cm in length) were extracted using a tissue protein extraction reagent (Thermo Fisher Scientific) with a protease inhibitor cocktail tablet (Roche) and 1 mM phenylmethylsulphonyl fluoride (PMSF). The amount of extracted protein was then quantified using bicinchoninic acid protein quantitative analysis. Proteins (40 mg/mL) were separated using sodium dodecyl sulfate-polyacrylamide gel electrophoresis and transferred onto a nitrocellulose blotting membrane (GE Healthcare, Freiburg, Germany). Blotting membranes were blocked using 3% BSA in TBST for 1 h at RT and incubated overnight at 4 ℃ with the primary antibodies. The membranes were washed and incubated with secondary peroxidase-conjugated antibodies for 1 h at RT on the following day. Protein expression was confirmed using enhanced chemiluminescence solution, which was exposed to LAS 4000 (BioRad, Hercules, CA, USA). Quantification of band intensity was performed using a multi-gauge V 3.0 software (Fujifilm, Tokyo, Japan). Full-length western blot images are presented in Additional file 7: Fig. S7.

#### Transmission electron microscope

Conventional transmission electron microscope (TEM) was used to observe the microarchitecture of axonal demyelination and degeneration in the injured SC specimens. Ultrathin Sects. (60–70 nm) of the injured SC specimens were obtained using an ultramicrotome (Leica Ultracut, Leica; Vienna, Austria) and double-stained with uranyl acetate and lead citrate before observation under TEM (JEM 1010, JOEL, Tokyo, Japan) at 60 kV.

#### Cell co-culture and assessment of in vitro neurite outgrowth

We used both two-dimensional (2D) and three-dimensional (3D) co-culture platforms to analyze neurite outgrowth during mature MN differentiation. For the co-culture, hMSC and iMN cells were cultured in a 1:1 ratio. In the 2D coculture platform, mature MN were differentiated for 10 days after seeding onto a laminin-coated plate. In the 3D co-culture platform, hMSC and iMN 3D aggregates were generated using microwell plates (AggreWell ^TM^ 800, STEMCELL Technologies, Seattle, WA, USA), in accordance with the manufacturer’s instructions. On day 2, hMSC and iMN aggregates were attached to the laminin-coated plate. After attachment of 3D spheroids, mature MN were induced for 10 days. Neurite outgrowth was evaluated using MAP-2 and neurite outgrowth assay kit (Life Technologies). Staining for relative neurite outgrowth was performed using the kit components and a background suppressor reagent, according to the manufacturer’s instructions. Relative neurite outgrowth was measured using a fluorescence plate reader to detect the fluorescence intensity from each well. Fluorescence was detected using emission settings at 554/567 nm for red fluorescent cell membrane staining.

#### Statistical analysis

Statistical analyses were performed using IBM SPSS Statistics for Windows, version XX (IBM Corp., Armonk, N.Y., USA). All results identified in the figures and text were expressed as mean ± SEMs. Statistical relevance was determined using one-way analysis of variance and Fisher’s least significant difference test (*). Kruskal–Wallis and Mann–Whitney (†) analyses were performed for intergroup comparison. Statistical significance was set at *p* < 0.05 (†* *p* < 0.05, ††** *p* < 0.01†††, *** *p* < 0.001, n.s = not significant).

## Results

### Human iPSC-derived iMNP and mature iMN generation

**Fig. 1:**
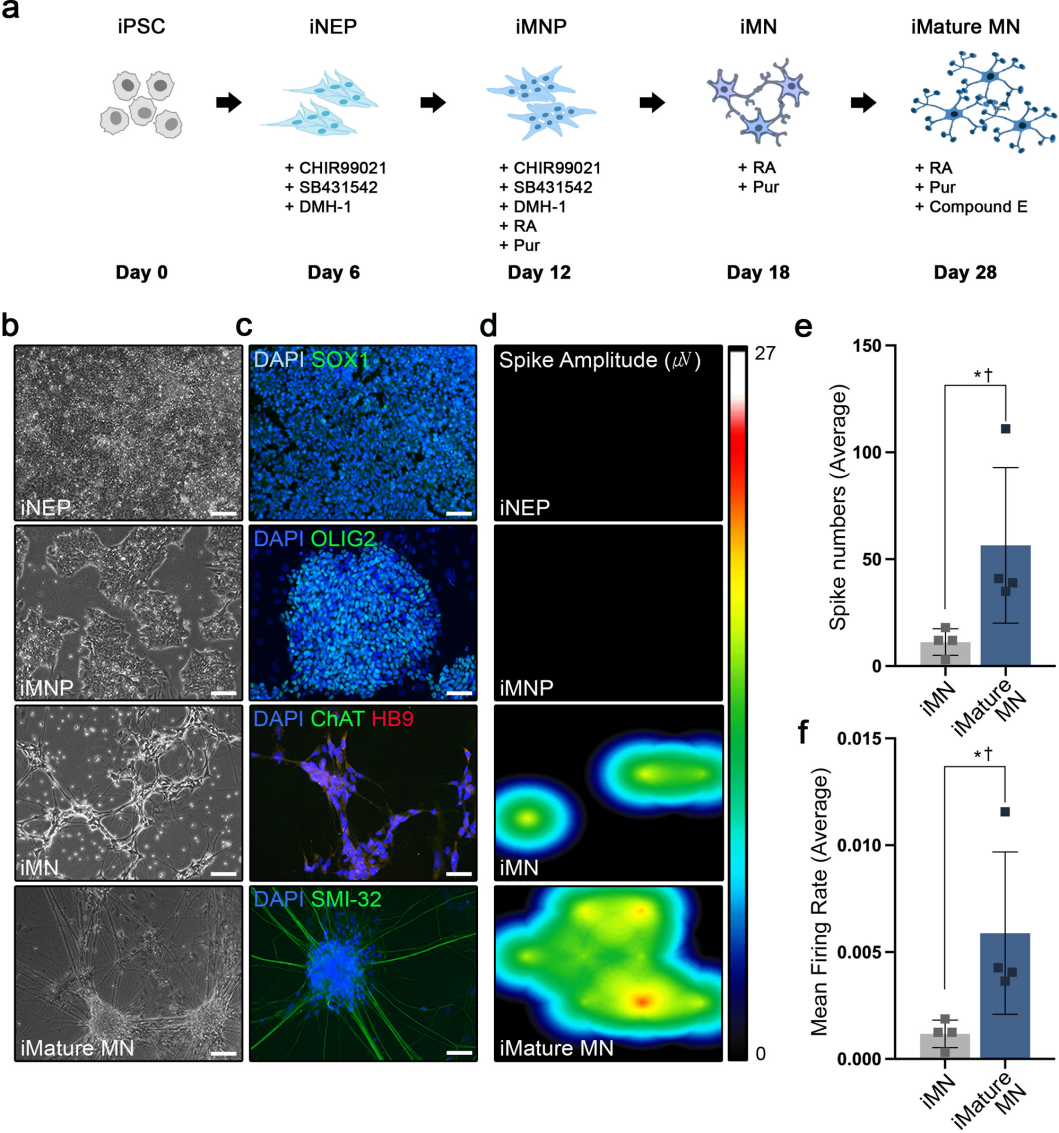
Generation of iMNP and iMN in vitro. **a** Scheme of MNP and MN differentiation from human iPSCs using a small molecule cocktail. **b** Representative time-course images of iNEPs after six days of culture media conditions, iMNPs on day 12 under different conditions with RA and Pur, and a representative image of iMNs on day 18. A light microscopy image showing the iMature MNs on day 28 of differentiation. **c** Fluorescence image showing the expression of SOX1 in iNEP, OLIG2 in iMNP, co-localization of ChAT and HB9 in iMN, and SMI-32 in mature iMN. **d** Representative images of heatmap activity for plate-wide visualization of spike or beat rates and amplitudes on multi-electrode arrays (iPSCs, *n* = 3; iNEP, *n* = 3; iMN, *n* = 4; iMature MN, *n* = 4). **e** Measure of average spike numbers of active electrodes per well. **f** Measurement of the mean firing rate of active electrodes per well over 28 days of in vitro differentiation. Data are presented as mean ± SEM. Statistical significance was estimated using Kruskal–Wallis test with post hoc analysis and Mann–Whitney (†) test with least significant difference post hoc analysis (*); *, † *P* < 0.05, Scale bars = 50 μm. iNEP, iPSC-derived neuroepithelial progenitor cells; iMNP, iPSC-derived motor neuron progenitor cells; iMN, iPSC-derived motor neuron cells; iMature MNs, iPSC-derived mature motor neuron cells

The generation of iMNP and iMN using a small-molecule cocktail was performed as previously described [19]. Our results confirm that iMNP and mature iMN were successfully generated and reproduced from human iPSCs (Fig. 1a). Neuro epithelial progenitor (iNEP) cells were generated from human iPSCs by treating small molecules including CHIR 99,021, DMH-1, and SB431542 for six days. iMNPs were obtained by treating the generated iNEPs with two additional supplements, RA and Pur, for an additional six days. Further six-day treatment with only RA and Pur produced iMNs from iMNPs. To induce iMN maturation, the cells were treated with RA, Pur, and Compound E for 10 days. Morphological changes were observed during differentiation (Fig. 1b). Successful differentiation was evaluated by confirming the expression of specific markers of each cell type (Fig. 1c). Differentiated iNEPs showed positive SOX1 expression. On day 12 of differentiation, majority of the differentiated iMNPs showed positive OLIG2 expression, and the co-expression of ChAT and HB9 in iMNs was confirmed on day 18 of differentiation. On day 28, most of the mature iMNs showed dramatic changes in morphology and tested positive for SMI-32. Multielectrode array (MEA) was used to confirm the electrophysiology of the generated mature iMNs. Neural spikes and activity were recorded, and immature and mature iMNs were found to be electrophysiologically active (Fig. 1d). MEA showed a higher number of spikes in iMature MNs than in iMNs (Fig. 1e). In addition, iMature MNs also showed a significantly increased level of electrophysiological properties compared to iMNs (Fig. 1f). Taken together, we confirmed the successful differentiation and maturation of MN generated from human iPSCs.

### Transplanted iMNPs induced MN maturation at the lesional site in chronic SCI

**Fig. 2:**
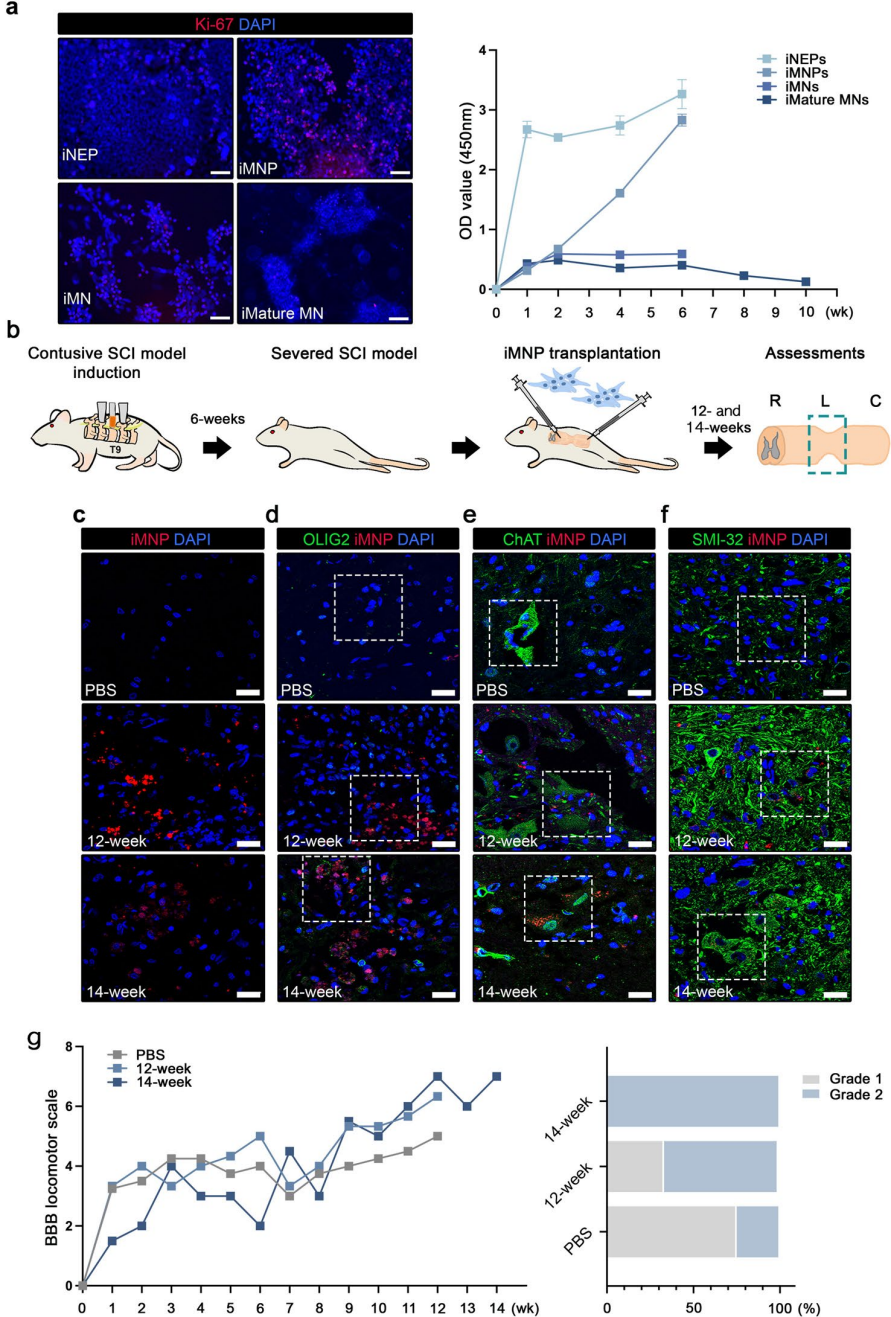
Transplantation of iMNP in contusive chronic SCI model. **a** Representative images of Ki-67 positive proliferating cells in each milestone cell type and proliferation were analyzed using the Cell Counting Kit-8 assay. **b** Experimental scheme of generation of contusive chronic SCI rat model. **c** The transplanted iMNPs show white and gray matter at the lesion epicenter. **d** IF stained image of OLIG2 and iMNP in the dorsal portion of the lesional site. **e** IF stained image of ChAT and iMNP in the gray matter of the lesional site. **f** Expression of SMI-32 in the transplanted iMNP cells was mostly noted around the ependymal cell layer and posterior part of the lesion. **g** BBB scores improved at 12 and 14 weeks after SCI (PBS, *n* = 4; 12 weeks iMNP, *n* = 3, and 14 weeks iMNP *n* = 2). Data are presented as mean ± SEM. Scale bars = 50 and 20 μm. iMNP, induced pluripotent stem cell-derived motor neuron progenitor cells; SCI, spinal cord injury; IF, immunofluorescence; ChAT, choline acetyltransferase; BBB, Basso–Beattie–Bresnahan

We attempted to confirm the therapeutic efficacy in injured SC of chronic SCI using differentiated cells (Subtopic 1). We first contemplated that the most proliferatively active cells may be beneficial as a therapeutic cell source that can lead to better engraftment and differentiation in vivo. Cell proliferation was confirmed in the milestone cell types during iMN differentiation, including iNEPs, iMNPs, iMNs, and iMature MNs. The results of Ki-67 staining and CCK8 assay confirmed that iMNPs had the highest proliferation rate (Fig. 2a). Therefore, iMNPs were selected for transplantation into the injured SC tissues. We conducted a pilot study to confirm the possibility of increased MN differentiation and maturation after iMNP transplantation into injured SC (Fig. 2b). Severed contusive chronic SCI was inflicted at T9 level using the Multicenter Animal Spinal Cord Injury Study (MASCIS) impactor. At six-weeks post injury, the injured site was re-exposed, and 1 × 10^6^ iMNPs were transplanted through IL injections. Engraftment and differentiation of MNs in the injured SC were assessed after 12–14 weeks. Transplanted iMNPs (PKH26 labeling, red color) at the lesional site were found along the IL injection route as clusters in the cystic and posterior gray commissure areas (Fig. 2c). To evaluate MN differentiation and maturation at the lesional site, expression of MNP cell marker OLIG2, and MN cell markers ChAT and SMI-32, was confirmed in the engrafted SC tissue. OLIG2 positive cells were found in the posterior cystic area (Fig. 2d). ChAT expression was confirmed in the cells near the posterior gray commissure area (Fig. 2e). SMI-32, a mature MN marker, was expressed near the posterior gray commissure area (Fig. 2f). Functional recovery was assessed using the BBB locomotor scale every week post injury. At 12- and 14-weeks post injury, the mean BBB locomotor scale values in the iMNP-transplanted groups showed slightly improved functional recovery compared to that in the PBS group. The groups were classified into grades based on the BBB locomotor scale scores, and the incidence ratio (%) was compared. In all groups, the ratio of achieving grade 3 functional recovery (BBB score 10–15) was 0%, whereas most achieved grades 1 (BBB score 0–5) and 2 (BBB score 5–10) (Fig. 2g). Based on the BBB scores, we attempted to identify the possible cause of limited in vivo recovery. A1 reactive astrocytes (C3) have been reported to be a possible barrier at the lesional site by previous studies [32–34]. Both Neurocan and GFAP expression indicated high expression of reactive astrocyte A1 type at the lesional site, which might have caused low engraftment in the injured gliosis scar in chronic SCI. Neurocan expression was confirmed in the cyst area (yellow dotted line) along with GFAP-positive cells (Additional file 1: Fig. S1a-b). Reactive astrocyte A1 type expended as complement component 3 (C3) was expressed in the cyst area. (Additional file 1: Fig. S1c). Based on the fluorescence intensity profile at the lesion site, we assumed that the decreased engraftment of the transplanted cells was caused by increased levels of Neurocan and reactive astrocyte (C3) expression (Additional file 1: Fig. S1d-f). As a result, possible MN differentiation and functional recovery induced by the engrafted cells at the lesional site was confirmed. However, the iMNP implant itself showed limitations in functional recovery.

### Multiple injections of human MSCs improved neuronal differentiation and neuroprotection after acute SCI

**Fig. 3:**
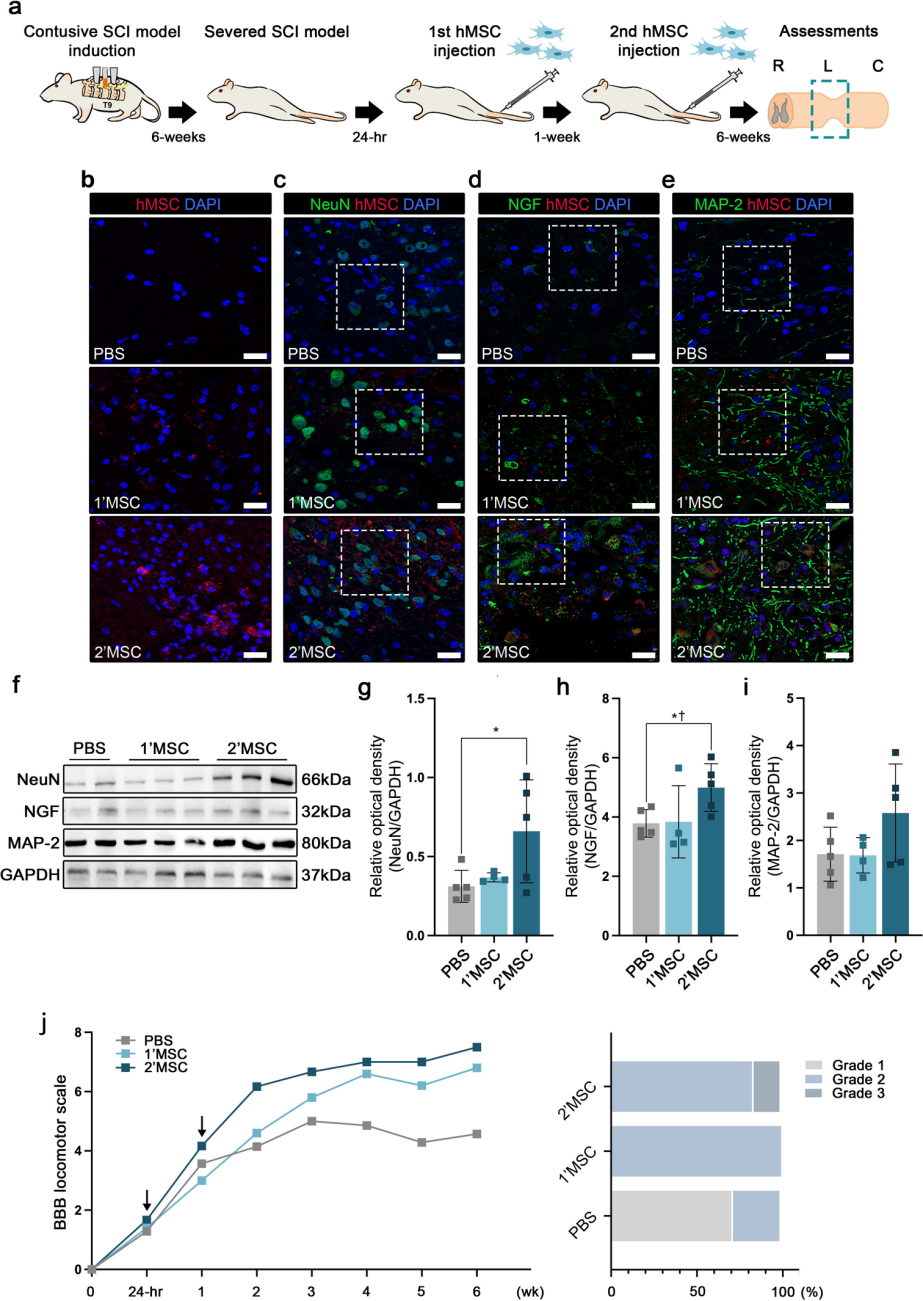
Induced neuronal differentiation and neuroprotection by multiple injections of hMSC at the lesional site. **a** Schematic of multiple intravenous injections of hMSC in contusive acute spinal cord injury (SCI). Animals were injected with hMSC (1’ MSC = single injection at 24 h post injury, 2’MSC = Dual injection at 24 h and one week post injury). **b** IF images show merged 4′,6-diamidino-2-phenylindole and hMSC at the lesional site at six weeks. **c** Multifluorescent, confocal images showing the expression of NeuN and hMSC in the gray matter. **d** Multi-fluorescent confocal images showing the expression of NGF and hMSC around the gray matter. **e** MAP-2 positivity indicates the lesional site and gray matter around and within hMSCs in trauma-induced gliosis at the posterior lesional site at six weeks. **f** WB images of NeuN, NGF and MAP-2 expression in lesional site segments ($\sim$ 1 cm). **g** WB results of NeuN expression in the lesional site segments. **h** WB results of NGF expression in the lesional site segments. **i** WB results of MAP-2 expression in the lesional site segments. Full-length western blot images are presented in Additional file 7: Fig. S7. **j** BBB scales in the 1’MSC and 2’MSC groups are significantly higher than that in the PBS group. Data are presented as mean ± SEM. Statistical significance was estimated using Kruskal–Wallis test with post hoc analysis and Mann–Whitney (†) test with least significant difference post hoc analysis (*); *, † *P* < 0.05. (WB analysis: PBS *n* = 5, 1’MSC *n* = 4 and 2’MSC *n* = 5, BBB locomotor scales: PBS *n* = 7, 1’MSC *n* = 5, 2’MSC *n* = 6) Scale bars = 20 μm. hMSC, human mesenchymal stem cells; IF, immunofluorescence; BBB, Basso–Beattie–Bresnahan; WB, Western blotting

Previous studies have reported that MSC transplantation via IV injection in acute SCI results in astrocytic and oligodendrocytic differentiation at the lesional sites [7, 8]. Based on previous reports, we attempted to promote the neuronal differentiation effect by administering IV injections of multiple hMSCs at acute SCI sites (Subtopic 2). IV injections of human MSCs (hMSCs) were administered 24 h and 1 week after acute SCI to improve the engraftment of transplanted cells into the severed SC (Fig. 3a). At six-weeks post injection, the transplanted hMSCs (PKH26 labeling, red color) were found in the injured SC in both single (1’MSC) and multiple (2’MSC) injected groups (Fig. 3b). Engraftment of hMSCs was observed around the cystic and posterior gray commissure areas. To confirm whether the transplanted cells differentiated into a neuronal lineage, expression of the neuronal marker NeuN was confirmed in the injured SC. The transplanted cells were positive for NeuN, mostly around the posterior gray commissure area, in both the injection groups (Fig. 3c). The 2’MSC group showed a higher expression of NeuN compared to the PBS and 1’MSC group (Fig. 3f and g). GFAP and CC-1 were expressed in the transplanted cells around the cystic area (Additional file 2: Fig.S2a-b). Moreover, GFAP and CC-1 expression in the 1’MSC group was higher in comparison to the PBS and 2’MSC group. However, no significant differences were observed among the groups (Additional File 2: Fig.S2c-d). We confirmed the increased C3 expression in the cystic area. However, C3 expression in the engrafted cells decreased in the injured SC. Western blot also showed a higher expression in the 1’MSC and 2’MSC groups than in the PBS group (Additional file 3: Fig.S3a-d). Nerve growth factor (NGF) expression was also increased in the posterior gray commissure area. We confirmed that NGF was strongly expressed around the engrafted cells; however, the transplanted hMSCs exhibited lower NGF expression in injured SC (Fig. 3d). Higher NGF expression was observed in the 2’MSC group compared to the PBS group, however, there was no significant difference with the 1’MSC group (Fig. 3f and h). The expression of BDNF and VEGF in the injured SC increased in the 2’MSC group; however, significant difference was not observed among the groups (Additional file 4: Fig.S4a-d). Axonal regeneration was assessed by MAP-2 expression at the site of injury. In the 2’MSC group, MAP-2 expression increased in the injured SC, however, significant difference was not observed among the groups (Fig. 3e and f, i). Functional recovery was assessed using BBB locomotor scales every week post injury. The mean BBB locomotor scale values in the 1’MSC and 2’MSC groups showed improved functional recovery compared to that in the PBS group at six-weeks post-injury, which was statistically significant. The 2’MSC group also showed a significantly improved BBB locomotor scale score compared to those in the PBS and 1’MSC groups at two-weeks post injury. However, significant difference was not observed between the 1’MSC and 2’MSC groups at six-weeks post injury. The incidence ratio of grade 3 (BBB score 10–15) in the 2’MSC group was 18.67% at six-weeks post injury (Fig. 3j).

### Stepwise cell therapy promoted mature iMN differentiation and behavioral improvement in severed SCI model

**Fig. 4:**
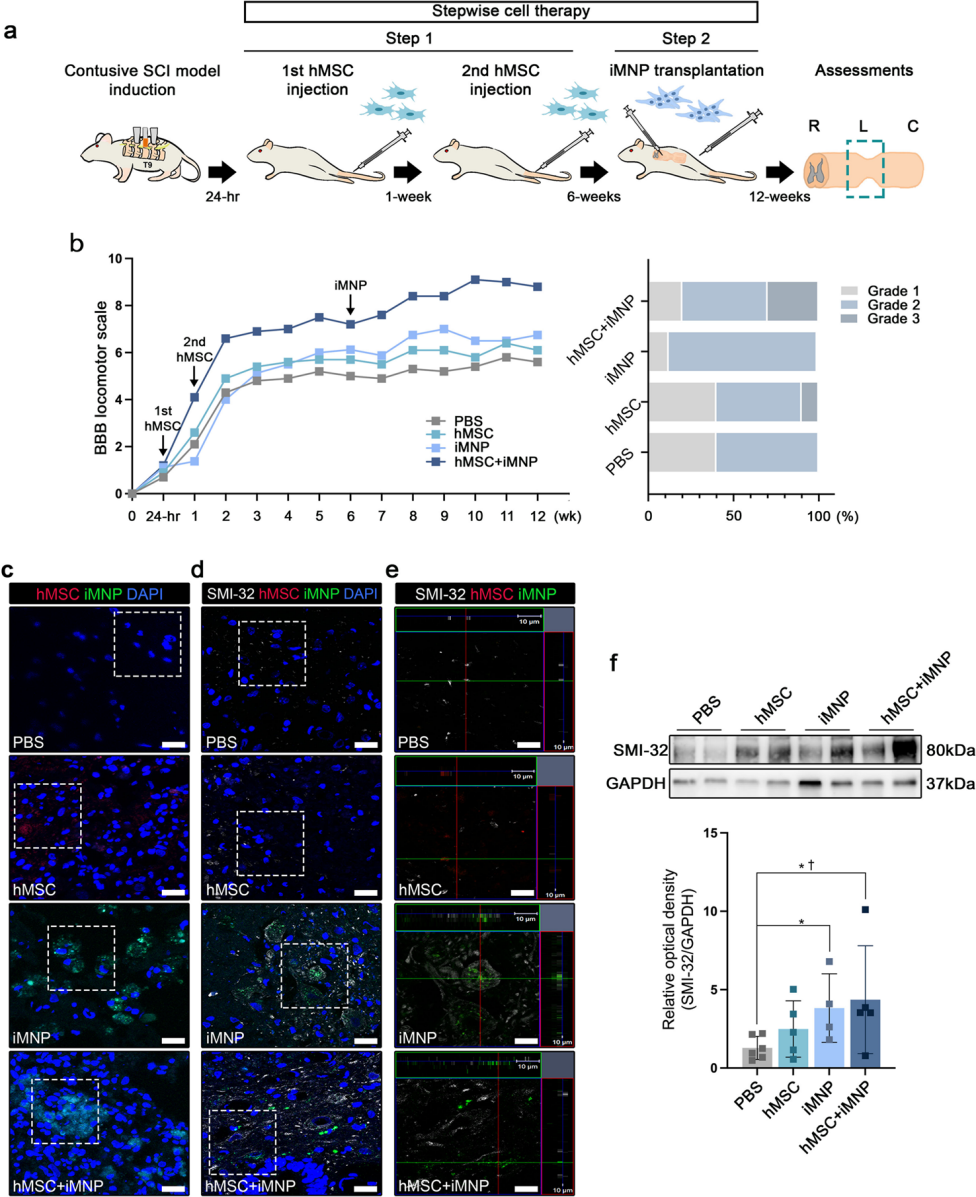
Enhancement of MN differentiation and maturation by preemptive cell transplantation of hMSC and iMNP in contusive SCI model. **a** Schematic image of preemptive transplantation of hMSC and iMNP in a contusive SCI model. Multiple intravenous injections of hMSC were administered at 24 h and one week post injury. Six weeks post injury, iMNPs were transplanted to the lesional site using intralesional injection. **b** BBB scale scores in the hMSC + iMNP group was significantly higher than that in the PBS group. **c** Transplanted hMSC and iMNP show white and gray matter at the epicenter of the lesion. **d** SMI-32 differentiation of transplanted iMNP cells is predominant. **e** Orthogonal view of confocal images showing SMI-32 and transplanted cell expression around the lesional site. **f** WB results of SMI-32 expression in the hMSC + iMNP group were significantly higher than those in the PBS group. Full-length WB images are presented in Additional file 7: Fig. S7. Data are presented as mean ± SEM. Statistical significance was estimated using Kruskal–Wallis test with post hoc analysis and Mann–Whitney (†) test with least significant difference post hoc analysis (*); *, † *P* < 0.05. (WB analysis: PBS *n* = 6, hMSC *n* = 5, iMNP *n* = 4 and hMSC + iMNP *n* = 5, BBB locomotor scales: PBS *n* = 10, hMSC *n* = 10, iMNP *n* = 8 and hMSC + iMNP *n* = 10) Scale bars = 20 and 10 μm. hMSC, human mesenchymal stem cells; iMNP, induced pluripotent stem cell-derived motor neuron progenitor cells; SCI, spinal cord injury; BBB, Basso–Beattie–Bresnahan; WB, Western blotting

We investigated how to enhance stem cell transplantation efficacy and MN differentiation in injured SC and confirmed the possible limitations of each cell type in vivo. To overcome these issues, we attempted a stepwise cell therapy strategy using both hMSC (PKH26 labeling, red color) and iMNP (PKH67 labeling, green color) (Main topic). First, hMSCs were injected 24 h and one-week post injury, and iMNP were delivered to the injured site at six-weeks post injury (Fig. 4a). At 12-weeks post injury, the mean BBB locomotor scale values in the hMSC + iMNP group showed significantly improved functional recovery compared to the PBS, MSC, and iMNP groups. The incidence ratio of grade 3 (BBB score 10–15) in the hMSC + iMNP group was 30% at 12-weeks post injury. In the severed SCI model, we confirmed that stepwise cell therapy increased the behavioral improvement incidence ratio compared to single-step cell therapy (Fig. 4b). In the hMSC and hMSC + iMNP groups, we confirmed that the transplanted hMSCs did not persist at the lesional site at 12-weeks post injury. In contrast, transplanted iMNPs persisted at the same time points (Fig. 4c). For the evaluation of MN differentiation in each group, SMI-32 expression was assessed. SMI-32 and PKH67 were highly expressed in the injured gray commissure area in the iMNP and hMSC + iMNP groups compared to the PBS and hMSC groups (Fig. 4d and e). The same trend was observed in the western blot analysis; however, no significant difference was observed between the iMNP and hMSC + iMNP groups (Fig. 4f). OLIG2 and ChAT expression levels were higher in the iMNP group than in the PBS, hMSC, and hMSC + iMNP groups. However, no significant differences were observed among the groups (Additional file 5: Fig. S5a-f).

### Stepwise cell therapy supported neural circuitry and axonal regeneration in the injured SC

**Fig. 5:**
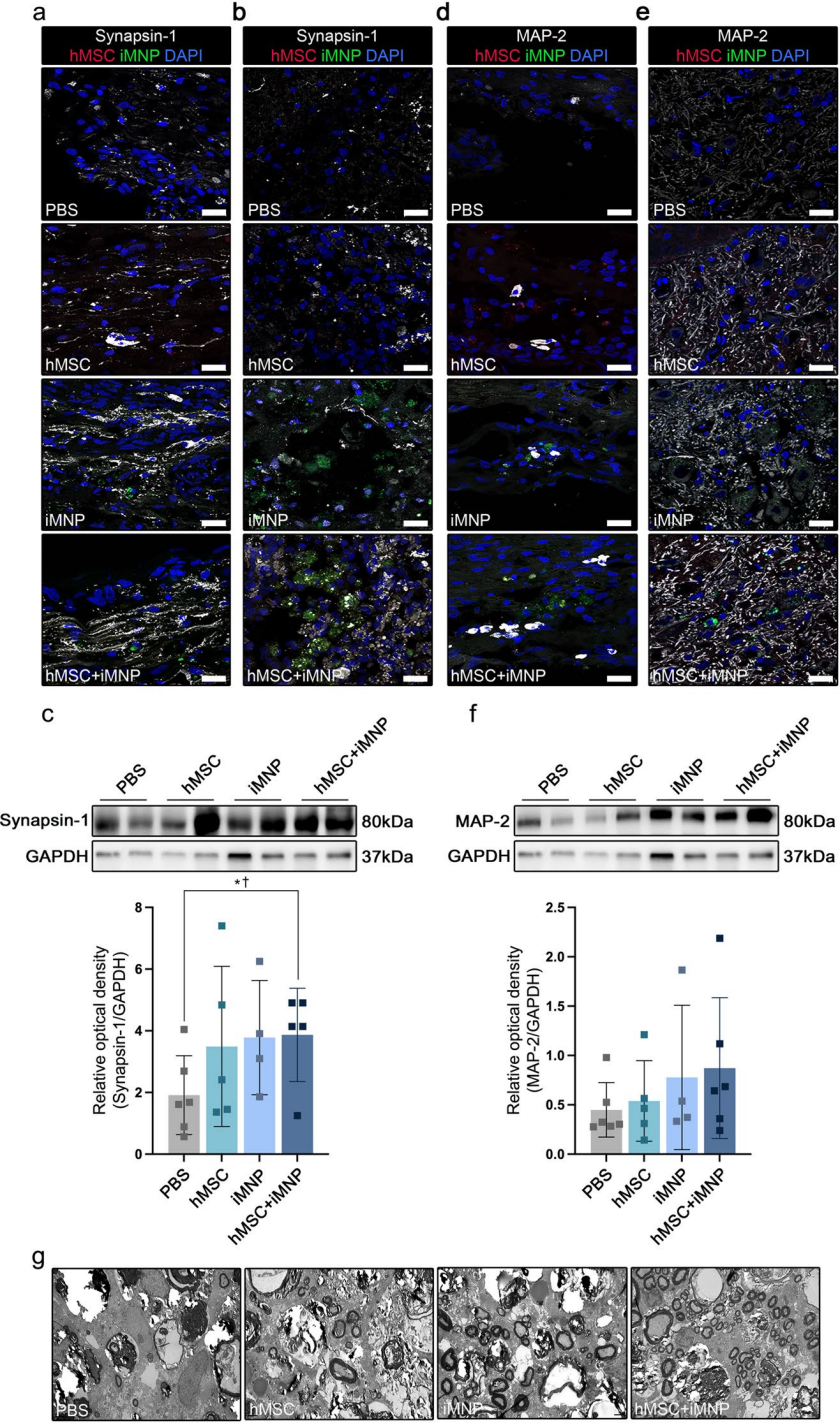
Increased axonal regeneration by preemptive hMSC and iMNP transplantation in the SCI lesion. **a** Confocal images showing synapsin-1 and transplanted cell expression around the lesional site. IF staining of synapsin-1 longitudinal sections. **b** IF staining of synapsin-1 showing axial sections of the lesional site. Synapsin-1 and transplanted iMNP merged around the lesional site. **c** WB results of synapsin-1 expression in lesional site segments ($\sim$ 1 cm). WB showed significantly increased expression of synapsin-1 in the MSC + MNP group compared to the PBS group. **d** IF staining images of MAP-2 showing a longitudinal section. **e** IF staining images of MAP-2 showing an axial section from the lesional site. MAP-2 and transplanted iMNP merged around the SCI lesional site. **f** WB results of MAP2 expression in the lesional site segments. Full-length WB images are presented in Additional file 7: Fig. S7. **g** Transmission electron microscopy images proximal to the lesional site. Data are presented as mean ± SEM. Statistical significance was estimated using Kruskal–Wallis test with post hoc analysis and Mann–Whitney (†) test with least significant difference post hoc analysis (*); *, † *P* < 0.05. (WB analysis: PBS *n* = 6, hMSC only *n* = 5, iMNP only *n* = 4, and hMSC + iMNP *n* = 5, Scale bars = 20 μm. hMSC, human mesenchymal stem cells; iMNP, induced pluripotent stem cell-derived motor neuron progenitor cells; SCI, spinal cord injury; IF, immunofluorescence; BBB, Basso–Beattie–Bresnahan; WB, Western blotting

Neural circuit formation and axonal regeneration are critical for the recovery of injured SC. Synapsin-1 expression was analyzed in the longitudinal and axial sections of injured SC at 12-weeks post injury. In the longitudinal section of injured SC, increased synapsin-1 expression was observed in the iMNP and hMSC + iMNP groups than in the PBS and hMSC groups. We confirmed that synapsin-1 was expressed around the engrafted iMNP at the lesional site (Fig. 5a and Additional file 6: Fig.S6a). In the axial section of injured SC, increased synapsin-1 expression was also observed in the iMNP and iMNP + hMSC groups (Fig. 5a and b). Western blot analysis showed that synapsin-1 expression was higher in the hMSC + iMNP group than in the PBS group; however, no significant difference was observed between the cell injection groups (Fig. 5c). Axonal sprouting was assessed by measuring MAP-2 expression in the longitudinal and axial sections of injured SC (Additional file 6: Fig. S6b). In the longitudinal and axial sections of the injured SC, MAP-2 expression was observed around the engrafted cells at the lesional site and in the posterior gray commissure area (Fig. 5d and e). MAP-2 showed a higher expression in the iMNP and hMSC + iMNP groups than in the PBS group (Fig. 5d and e). The same trend was observed by western blotting; however, the difference was not significant (Fig. 5f). Ultrastructural changes in the injured SC were analyzed using TEM. At 12-weeks post injury, increased myelination was observed in the myelin sheath of injured SC in the hMSC + iMNP group. In contrast, the PBS, hMSC, and iMNP groups showed increased axonal degeneration in the myelin sheath collapse in the injured SC (Fig. 5g).

### hMSCs and iMNs acted synergistically to promote MN neurite outgrowth induction in vitro

**Fig. 6:**
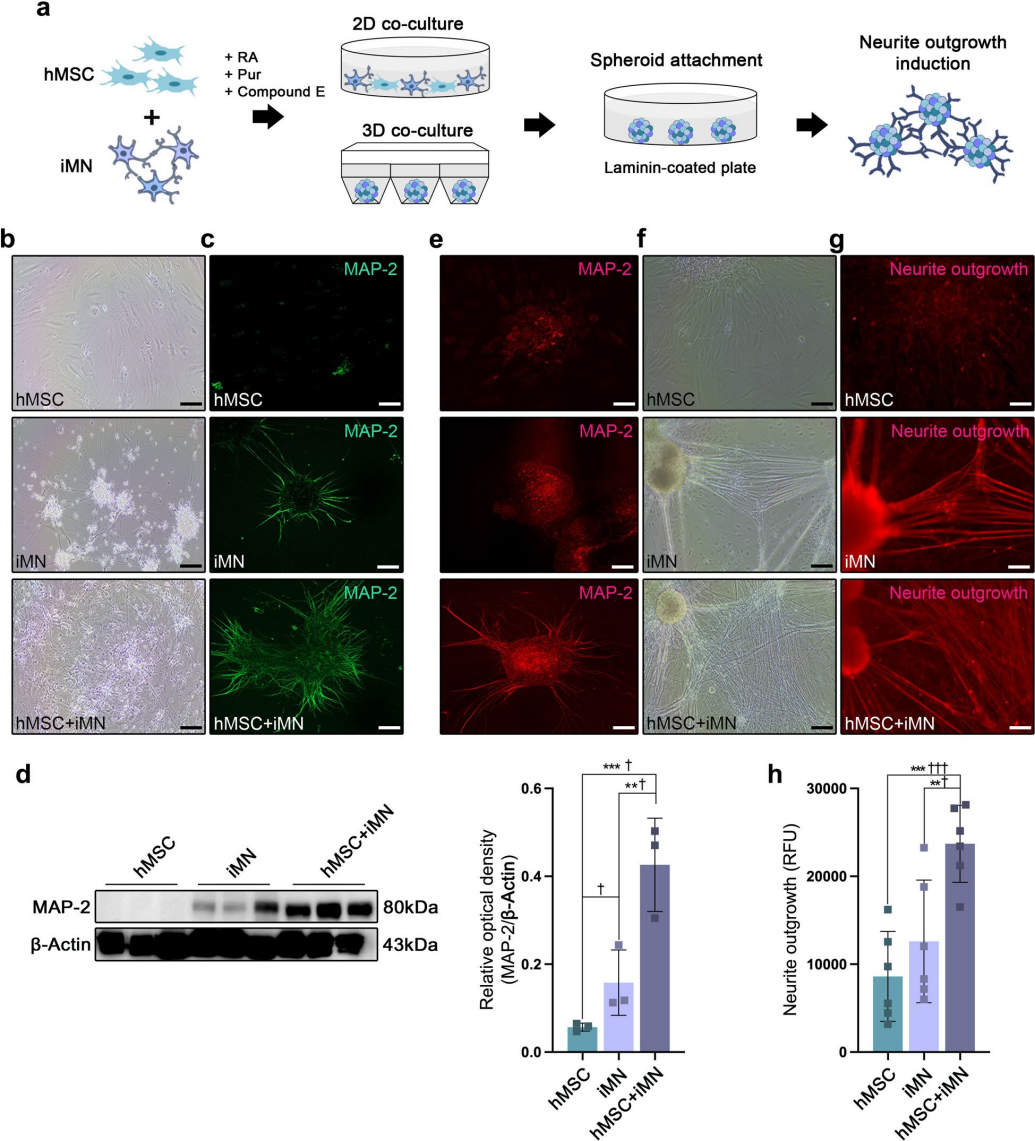
Neurite outgrowth induction by 2D and 3D co-culture of hMSC and iMN in vitro. **a** Schematic of hMSC and iMN 2D and 3D co-culture neurite outgrowth assessment in vitro. **b** Representative light microscopy images of hMSC and iMN 2D co-culture into mature MN differentiation on day 10. **c** IF staining images of MAP-2 in 2D co-cultured hMSC and iMN on day 10. **d** Protein expression of MAP-2 in hMSC and iMN 2D co-culture cell lysates on day 10. Full-length WB images are presented in Additional file 7: Fig. S7. **e** IF staining of MAP-2 in 3D co-cultured spheroids consisting of hMSC and iMN on day 10. **f** Representative light microscopy images of neurite outgrowths on day 10 in the 3D co-cultured spheroids. **g** Representative fluorescence microscopy images of neurite outgrowths on day 10 in the 3D co-cultured spheroids. **h** Quantification of neurite outgrowth on day 10. Data are presented as mean ± SEM. Statistical significance was estimated using Kruskal–Wallis test with post hoc analysis and Mann–Whitney (†) test with least significant difference post hoc analysis (*); *, † *P* < 0.05. (WB analysis: hMSC *n* = 3, iMN *n* = 3, hMSC + iMN *n* = 3, Neurite outgrowth assay analysis: hMSC *n* = 6, iMN *n* = 6, hMSC + iMN *n* = 6, Scale bars = 20 μm. hMSC, human mesenchymal stem cells; iMN, induced pluripotent stem cell-derived motor neuron cells; IF, immunofluorescence; WB, Western blotting

We showed that stepwise combined cell transplantation using hMSC and iMNP increased axonal regeneration in injured SC; however, it was difficult to confirm the possible mechanism in vivo. We hypothesized that the combination of hMSCs and iMNs would promote the induction of neurite outgrowth during MN differentiation and maturation. To confirm this hypothesis, we analyzed the induction of neurite outgrowth by co-culturing hMSCs and iMNs on both 2D and3D spheroid platforms during mature iMN differentiation (Fig. 6a). Neurite outgrowths were observed at day 10 of differentiation in 2D co-cultured hMSC and iMN (Fig. 6b). Neurite outgrowth was assessed in the hMSC + iMN 2D co-culture using MAP-2 on day 10, and qualitatively increased neurite outgrowth was observed in the hMSC + iMN group compared to that in the hMSC and iMN groups (Fig. 6C). Western blot analysis showed that MAP-2 expression was significantly higher in the hMSC + iMN group than in the hMSC and iMN groups (Fig. 6d). MAP-2 expression was also confirmed in co-cultured hMSC and iMN 3D spheroids on day 10 of differentiation (Fig. 6e). We attached 3D spheroids to laminin-coated plates for neurite outgrowth assay analysis and observed neurite outgrowth on day 10 (Fig. 6f). Neurite outgrowth of the 3D spheroids was assessed using a neurite outgrowth staining kit, and it was confirmed that neurite outgrowth was significantly higher in the hMSC + iMN group than in the hMSC and iMN groups (Fig. 6g and h).

## Discussion

SCI is divided into traumatic SCI (TSCI) and nontraumatic SCI [35]. Causes of TSCI include automobile accidents, falls, work-related injuries, violent crimes, and sports-related injuries [3]. TSCI has physical, psychological, and occupational effects on patients [36]. The pathophysiology of SCI is characterized by a primary mechanical injury to the SC followed by a secondary cascade of cellular and molecular events that further propagate the injury to SC tissue. Primary injuries can occur through various mechanisms, including compression, contusion, transection, and shearing forces on the SC. After a primary injury to the SC occurs, a secondary injury event is initiated in the injured SC tissue, which expands the area of neural tissue injury and exacerbates neurological deficits and outcomes [3, 37, 38]. Contusive SCI causes immediate mechanical damage due to permanent or temporary compression of the SC, which is followed by increasing functional impairment over hours and weeks. Moreover, it also leads to a secondary injury characterized by biochemical changes that increase the number of glial and microglial cells, resulting in cell death at the lesional site [38–40]. Pre-clinical research on SCI is being conducted using various animal models. The rat model of SCI is an important mammalian model for evaluating the pathological basis of SCI and its therapeutic strategies [41, 42]. The contusive SCI rat model serves as a suitable model for developing and evaluating the structural and functional benefits of treatment strategies for SCI, which results from transient physical impact to the SC and is clinically relevant [43]. Based on previous studies, we confirmed the possibility of stem cell therapy using a rat model of contusive SCI.

Secondary SCI can be categorized into acute, subacute (or intermediate), and chronic phases depending on the time elapsed after injury and the pathological mechanism. Acute SCI phase is expressed in the last 48 h after the initial physical insult to the SC. The main phenomena observed in the acute SCI phase are vascular disruption, hemorrhage, and subsequent ischemia of the injured SC. After disruption of the microenvironment in the injured SC, pathological changes such as ion dysregulation, excitotoxicity, excessive production of free radicals, and inflammatory responses cause further damage to the neurons and glial cells at the lesional site [3, 44, 45]. The sub-acute (intermediate) phase is considered to last up two weeks in the injured SC. Characteristic features of the subacute phase are phagocytic response and reactive proliferation of astrocytes at the lesional site. Increase in the number of reactive astrocytes at the lesional site results in the formation of a glial scar. Glial scarring is a major cause of limited neuroregeneration and axonal regeneration in SCI [3, 46, 47]. A characteristic feature of chronic SCI phase is the maturation of the lesion, including scar formation and development of a syrinx at the lesional site. Endogenous regenerative capacity is affected by the release of growth-inhibitory molecules and glial scarring around the epicenter of the lesion [3, 44]. 

Neuroprotective and neuroregenerative approaches are being used to treat SCI [3]. Transplantation of various types of stem cells reportedly has great potential to preserve damaged tissues and promote the recovery of nerve function [46, 48]. Previous studies have reported that bone marrow-derived MSC (BM-MSC) transplantation in acute and chronic SCI rat models increased clinical improvement, neuroprotection, and neuroregeneration via differentiated astrocytes and oligodendrocytes at the lesional sites [7–9, 27]. The findings of the aforementioned studies on transplantation of BM-MSCs in SCI suggests the potential for cell therapy in acute and chronic SCI models. It has been shown that the densities of astrocytes and oligodendrocytes return to near-normal levels in the residual white matter within several weeks after SCI. However, chronic SCI causes loss of gray matter neurons due to damage at the lesional site and permanent damage to motor function. In chronic SCI, a disorder characterized by progressive loss of motor neurons, a feasible treatment approach is to replace lost or degenerated motor neurons [20]. 

Recently, high-purity iMNPs and iMNs were successfully established, and their transplantation in SCI was studied [19, 30]. Another study reported that transplantation of human embryonic stem cell (hESC)-derived motor neurons progenitor (MP) enhanced astrogliosis at the lesional site four-months after SCI. In addition, it was confirmed that increased astrogliosis at the lesional site favored the survival and differentiation of hESC-derived neurons and correlated with improved motor function recovery [21]. In another study, when a high-purity population of human motor neuron progenitors (hMNP) derived from hESC was transplanted into an SCI rat model, it was reported that hMNP at the lesional site suppressed the intracellular signaling pathway associated with SCI pathogenesis, which correlated with greater endogenous neural survival and neurite branching [22]. However, studies on their therapeutic effects in SCI are lacking. We aimed to confirm the increased differentiation of gray matter neurons and recovery of motor function at the lesional site by transplantation of iPSC-derived motor neuron progenitor cells (iMNP) in a chronic SCI model. In the context of subtopic 1 of the study, we confirmed the efficacy of iMNP in neuroregeneration in a chronic SCI model. We generated iPSC-derived iMNPs and iMNs in vitro and selected iMNPs with high proliferation for successful transplantation into the SC of chronic SCI model (Fig. 2a and b). The iMNPs transplanted at the lesional site showed possible in vivo MN differentiation and maturation. Moreover, the cells also showed behavioral recovery through BBB locomotor scale scores (Fig. 2c-g). Our findings suggest successful engraftment and MN differentiation of implanted iMNP in chronic SCI. However, the subsequent formation of glial scars and their microenvironment could not be effectively decreased at the injured site. In addition, the transplanted iMNP did not decrease the expression of reactive gliosis in the round cystic area in chronic SCI (Additional file 1: Fig.S1a-g). However, subtopic 1 of the study had a limitation in that few animals were used due to the pilot study concept.

Transplantation in chronic SCI results in low rate of engraftment and functional restoration due to the subsequent formation of glial scars [8, 29]. Therefore, the timing of transplantation must be taken into consideration and alterations in the microenvironment of chronic SCI tissues should be induced to enhance the effectiveness of transplantation [29, 49]. MSC transplantation can prevent the secretion of various inflammatory cytokines, apoptosis, and inflammation to exert neuroprotection during acute SCI [7, 50–52]. Based on the results of previous studies and our initial findings, we attempted to increase the transplantation effect at the lesional site through multiple hMSC injections for acute SCI (Subtopic 2), and found that it increased the cell transplantation efficacy in comparison to single hMSC injection by increasing neuronal cells via NGF and axonal regeneration at the lesional site (Fig. 3f and g). Interestingly, multiple hMSC injections promoted clinical recovery by increasing neuronal cell differentiation, whereas a single hMSC injection promoted clinical recovery by increasing astrocyte and oligodendrocyte differentiation in the injured SC (Additional file 2: Fig.S2a-b). C3 is a specific marker of A1 neuroinflammation-reactive astrocytes in SCI, and evaluation of the phenotype of reactive astrocytes in SCI have been reported by several studies [23, 32, 33, 53]. One research reported that an IV injection of MSC-derived exosomes reduced the number of C3- or GFAP-positive astrocytes at the lesional site in acute SCI [53]. Our findings suggest that multiple and single injections of hMSCs can effectively promote functional behavioral recovery by decreasing C3 expression at the lesional site in acute SCI (Additional file 3: Fig.S3a-c).

Stem cell-derived MN and MNP offer promising strategies for cellular replacement in SCI. However, in our pilot experiment, the data suggested that iMNP cell transplantation confirmed the limitations of cellular replacement strategies in chronic stage astrocyte and Neurocan formation scar cavity. Thus, we used a stepwise cell therapy strategy for SCI to increase the efficacy of the transplanted cells at the lesional site. This main topic of the study included confirmation of the neuroprotective and neuroregenerative effects of multiple preemptive hMSC injections and increased MN differentiation at the lesional site through transplanted iMNP. Interestingly, we found that stepwise cell therapy promoted MN maturation and axonal regeneration at the lesional site. We also confirmed that cell transplantation with iMNP alone increased MN differentiation at the lesional site (Additional file 4: Fig.S4a-d). Another study reported that MNP cell transplantation resulted in MN lineage differentiation in the ventral horns at the lesional site. However, the failure of MNPs to mature in all other regions of the SC likely reflected the gliogenic nature of the SCI environment [22]. Our findings suggest that the increased neuroprotective effects of multiple preemptive hMSC injections in the acute SCI phase can enhance MN differentiation and maturation at the lesional site. More importantly, it was demonstrated in vitro that hMSC and iMN co-culture significantly increased neurite outgrowth during the MN maturation stage (Fig. 6b-g). Another study reported that differentiated Schwann cells (SC), human bone marrow-MSCs, and umbilical-cord-blood MSCs significantly promoted neurite outgrowth and elongation in comparison to untreated MSCs [54]. We found that 2D and 3D co-cultured hMSC and iMN induced neurite outgrowth and elongation compared to hMSC and iMN separately (Fig. 6f-g). However, hMSC alone did not significantly promote neurite outgrowth and elongation as compared to iMN. Another study suggested that hMSC promotes neurite outgrowth via a paracrine effect through growth factors including BDNF and NGF [55–57]. 

In summary, this study confirmed that clinical behavioral outcomes were restored through induction of mature motor neuron differentiation and axonal regeneration at the lesional site using stepwise combined cell transplantation of hMSCs and iMNPs in a contusive SCI model, suggesting the therapeutic efficacy of stepwise combined cell transplantation strategy in a severed contusion SCI rat model. The stepwise combined cell transplantation strategy has the advantage of not only suggesting ideal stem cell selection for each stage of SCI, but also confirming the function of the transplanted cells. However, a limitation of this study is the lack of an explanation for the mechanisms underlying the synergistic effect of stepwise combined cell transplantation in a contusion SCI model. In future studies, it will be necessary to confirm the synergistic effects of stepwise combined cell transplantation mechanisms using time-dependent RNA sequencing (RNA-seq) or single-cell analysis at the lesional site. In addition, selecting a sample size for animal experiments requires calculating a sample size sufficient for statistical analysis using a few free software packages (G power, power sample size). There is a need to overcome the limitations of stem cell therapy for SCI using a stepwise combined cell transplantation strategy with a 3D iPSC-derived motor neuron source.

## Conclusion

Our study demonstrated that stepwise cell therapy increased MN differentiation and axonal regeneration compared to single-cell therapy in severed SCI model. Stepwise cell therapy increased behavioral recovery and the rate of BBB locomotor scale grade 3 (BBB score, 10–15). Moreover, it also induced alterations in the microenvironment for effective cell therapy in severed SCI model. These in vitro results suggest that co-cultured hMSC and iMN synergistically promoted induction of MN neurite outgrowth. Taken together, we report a proof-of-concept study showing that stepwise combined transplantation can increase cell engraftment and SC recovery based on cell type and transplantation timing in SCI.

### Electronic supplementary material

Below is the link to the electronic supplementary material.

Supplementary Material 7

Supplementary Material 1

Supplementary Material 2

Supplementary Material 3

Supplementary Material 4

Supplementary Material 5

Supplementary Material 6

Supplementary Material 8

## Data Availability

All datasets generated during the study are included within the article.

## Abbreviations

AVMA: American veterinary medical association
ARRIVE: Animal research: reporting of in vivo experiments
BBB: Basso–Beattie–Bresnahan
BSA: Bovine serum albumin
BDNF: Brain-derived neurotrophic factor
CCK-8: Cell counting kit-8
C3: Complement component 3
ChAT: Choline acetyltransferase
DMEM: Dulbecco’s modified eagle’s medium
DMH1: Dorsomorphin homologue 1
DAPI: 4′,6-diamidino-2-phenylindole
ESC: Embryonic stem cell
GFAP: Glial fibrillary acidic protein
hMSC: human mesenchymal stem cells
iPSC: Induced pluripotent stem cell
iNEP: Induced pluripotent stem cell derived Neuro epithelial progenitor
iMNP: Induced pluripotent stem cell derived motor neuron progenitor cells
iMN: Induced pluripotent stem cell derived motor neuron
iMature MNs: Induced pluripotent stem cell derived mature motor neurons
IL: Intra lesional
IV: Intra venous
MEA: Multi-electrode array
MNs: Motor neuron cells
MASCIS: Multicenter animal spinal cord injury study
NSPCs: Neural stem and progenitor cells
NGF: Nerve growth factor
OECs: Olfactory ensheathing cells
OCT: Optimal cutting temperature
PBS: Phosphate-buffered saline
PBMCs: Peripheral blood mononuclear cells
PFA: Paraformaldehyde
PMSF: Phenyl methyl sulfonyl fluoride
Pur: Pumorphamine
RT: Room temperature
RA: Retinoic acid
SCI: Spinal cord injury
SC: Spinal cord
SD: Sprague–Dawley
TEM: Transmission electron microscopy
TBS: Tris-buffered saline
TBST: tris-buffered saline with 0.05% Tween-20
VEGF: Vascular endothelial growth factor
