# Supplementary material for: Stepwise combined cell transplantation using mesenchymal stem cells and induced pluripotent stem cell-derived motor neuron progenitor cells in spinal cord injury
Source: Stem Cell Res Ther. 2024 Apr 23;15:114. doi: 10.1186/s13287-024-03714-3 (PMC11036722; doi:10.1186/s13287-024-03714-3)

Full-length western blot images for fig.3 f-h

Neu N 60kDa

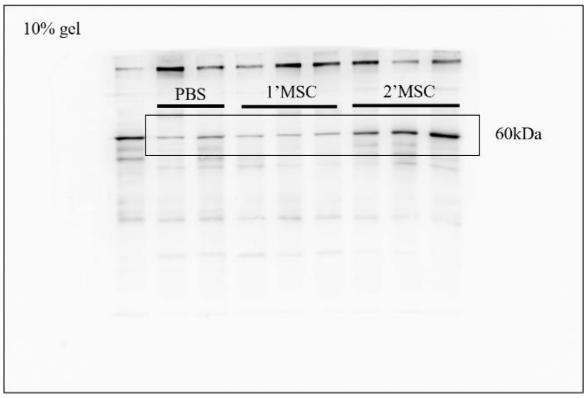

NGF 32kDa

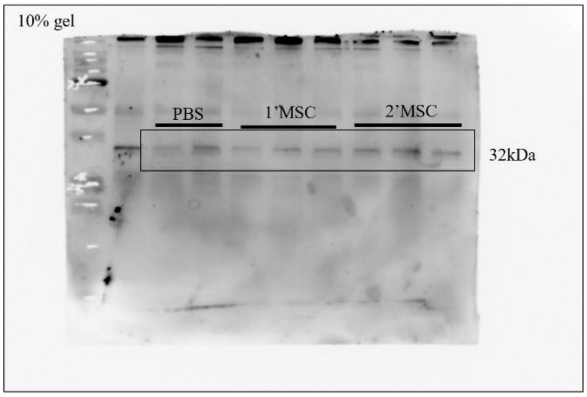

MAP-2 80kDa

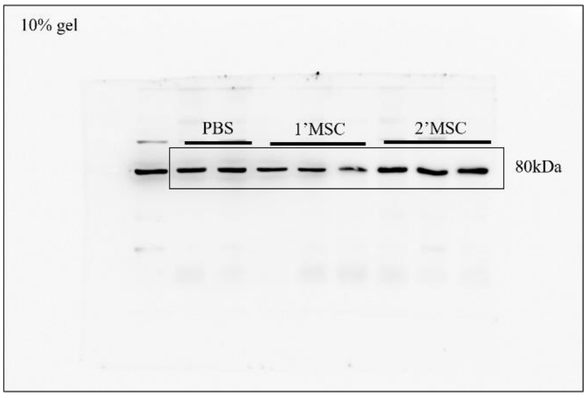

GAPDH 37kDa

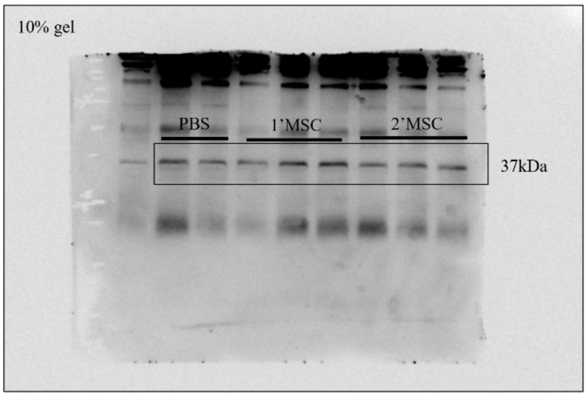

Full-length western blot images for fig.4 f

SMI-32 70kDa

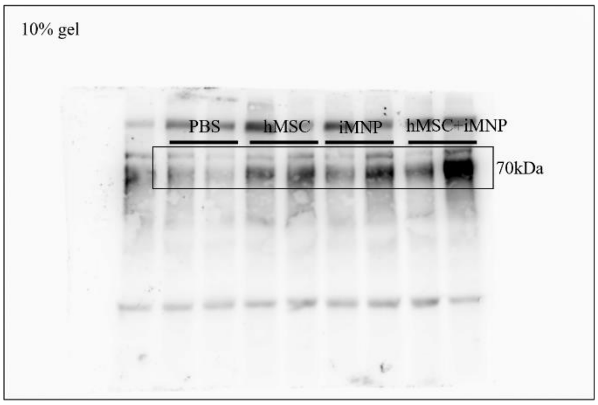

GAPDH 37kDa

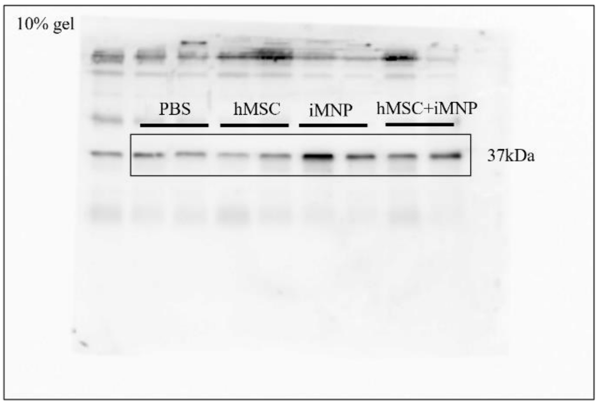

Full-length western blot images for fig.5 c and f

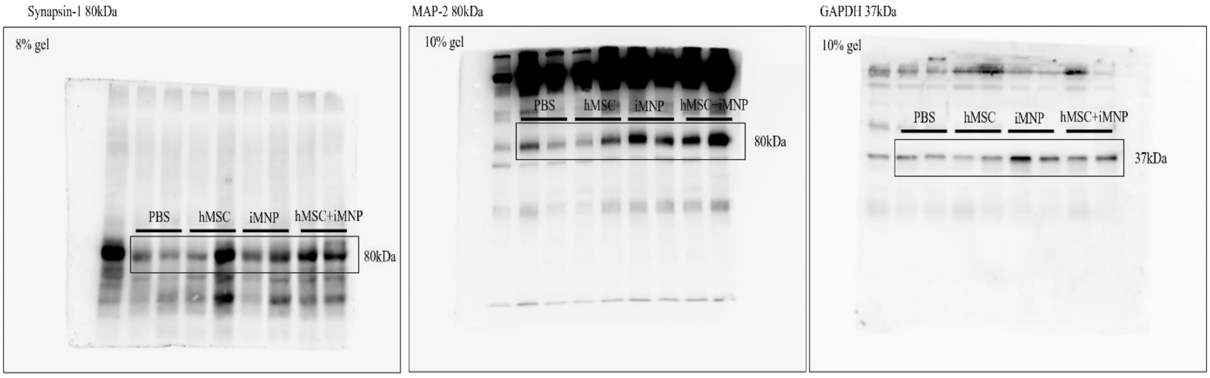

Full-length western blot images for fig.6 d

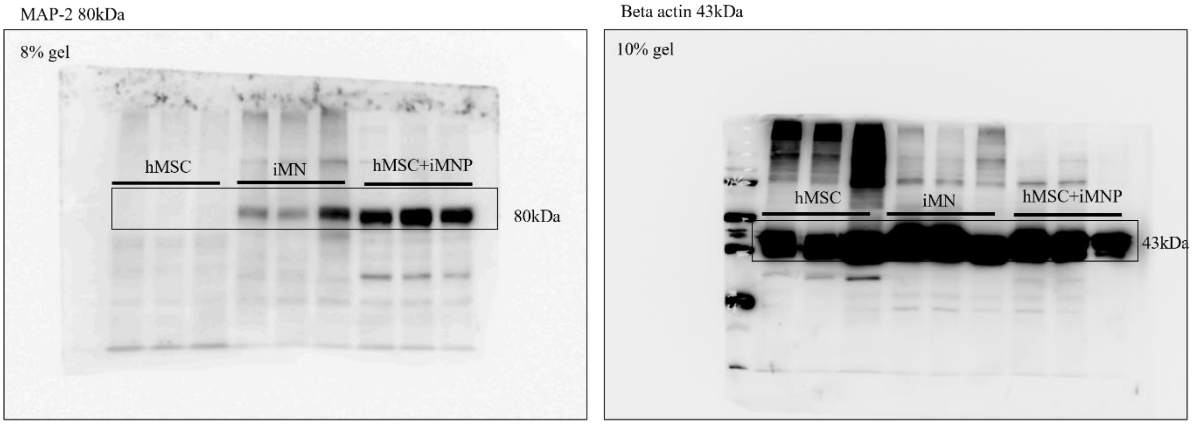

Full-length western blot images for additional file 2: Fig S2 c and d

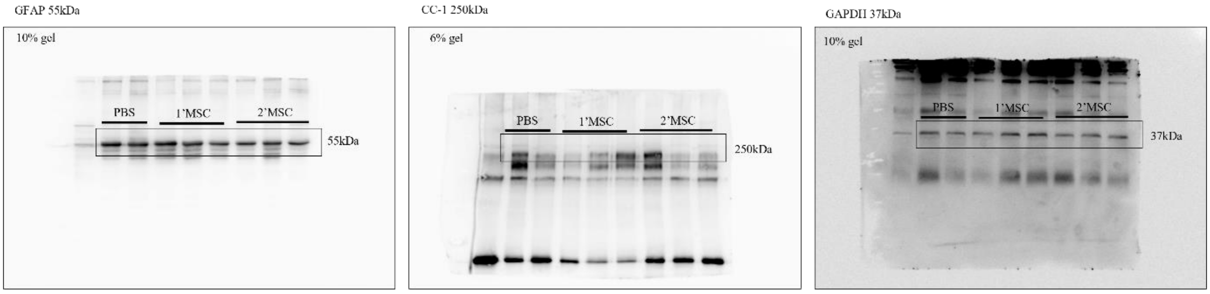

Full-length western blot images for additional file 3: Fig S3 c

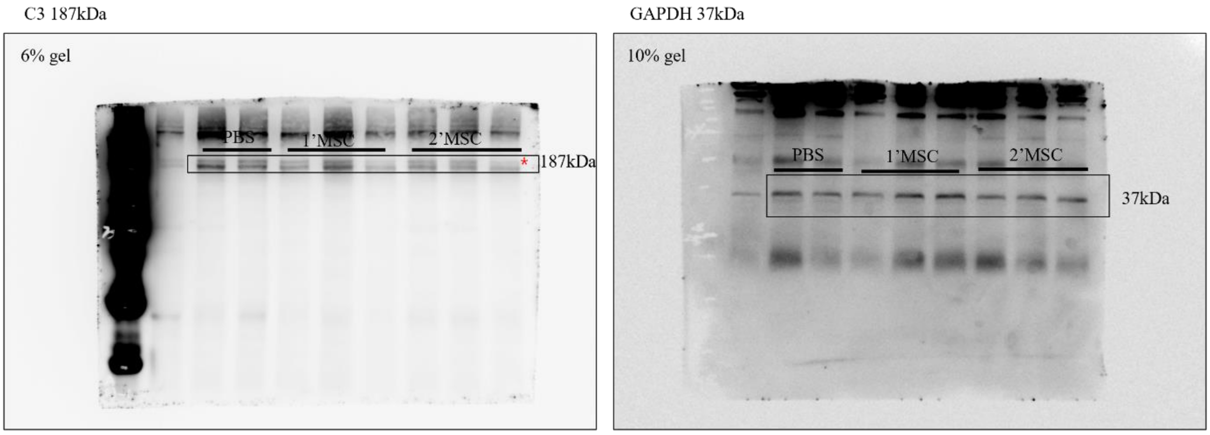

Full-length western blot images for additional file 4: Fig S4 c and d

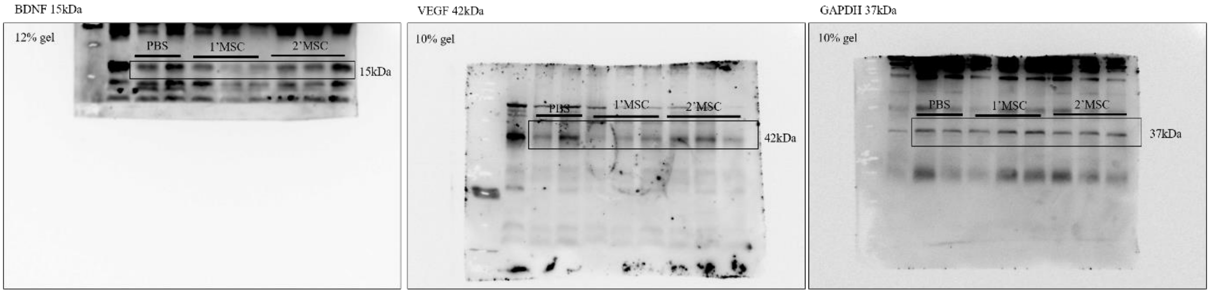

Full-length western blot images for additional file 5: Fig S5 e and f

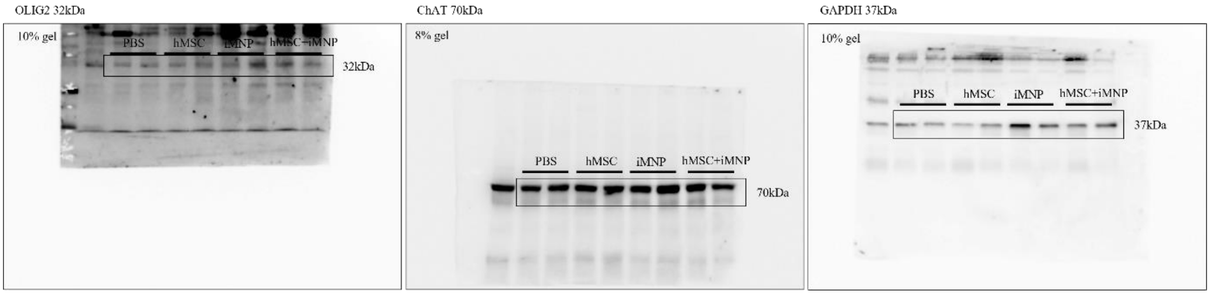

Supplement: Supplementary file 1 — Supplementary Material 7 [file 13287_2024_3714_MOESM1_ESM.pdf]
