# Supplementary figures and images for: Stepwise combined cell transplantation using mesenchymal stem cells and induced pluripotent stem cell-derived motor neuron progenitor cells in spinal cord injury
Source: Stem Cell Res Ther. 2024 Apr 23;15:114. doi: 10.1186/s13287-024-03714-3 (PMC11036722; doi:10.1186/s13287-024-03714-3)

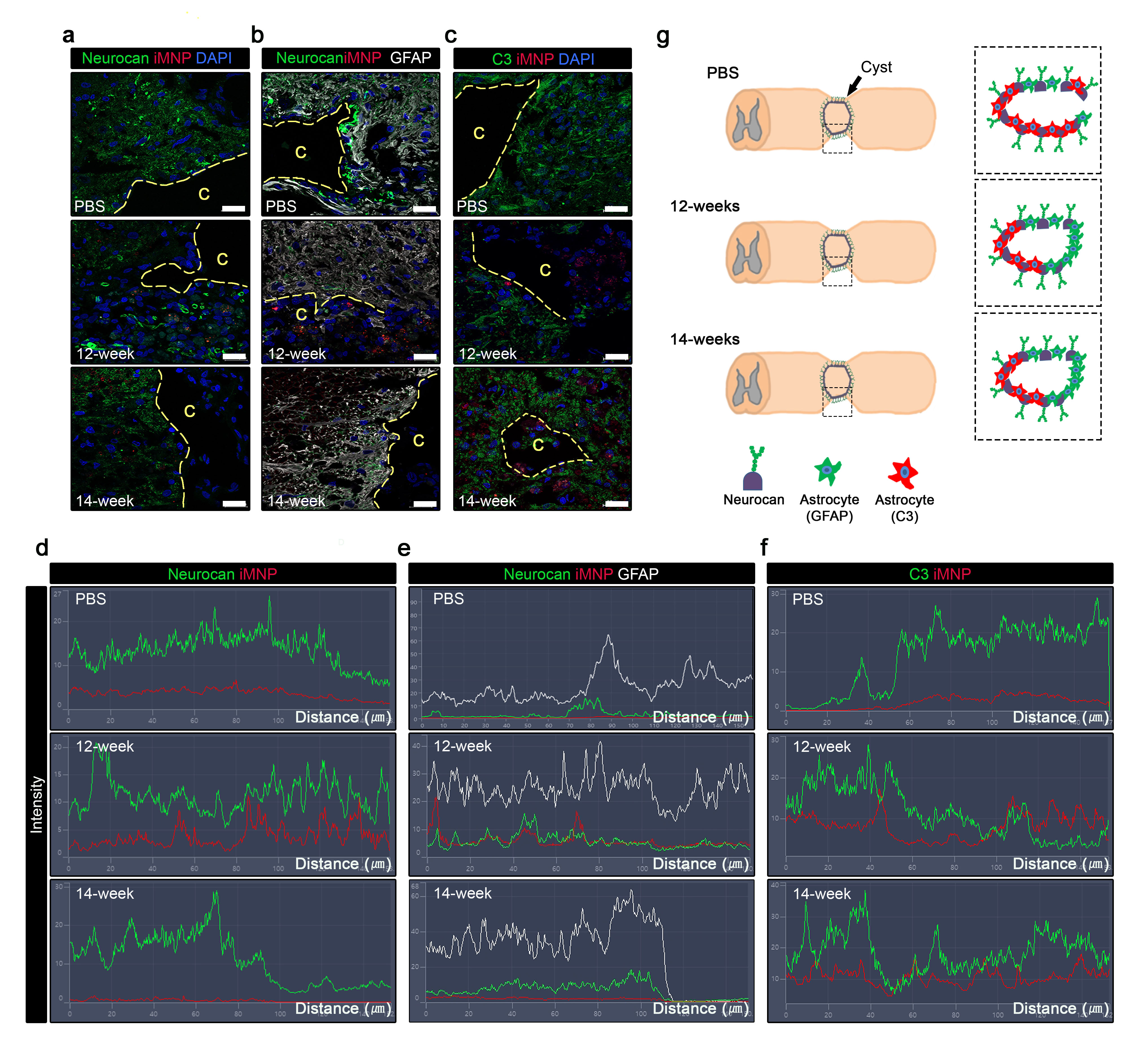

Supplement: Supplementary file 2 — Supplementary Material 1 [file 13287_2024_3714_MOESM2_ESM.jpg]

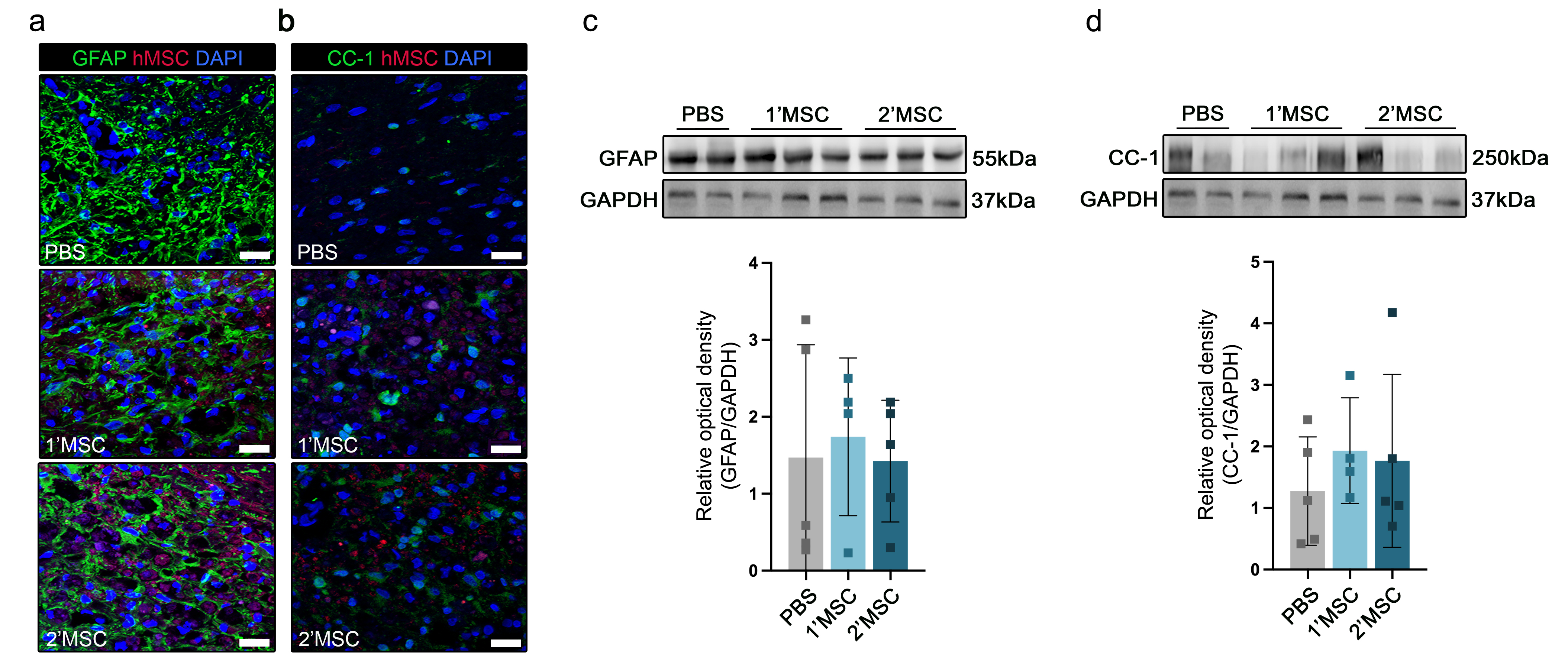

Supplement: Supplementary file 3 — Supplementary Material 2 [file 13287_2024_3714_MOESM3_ESM.jpg]

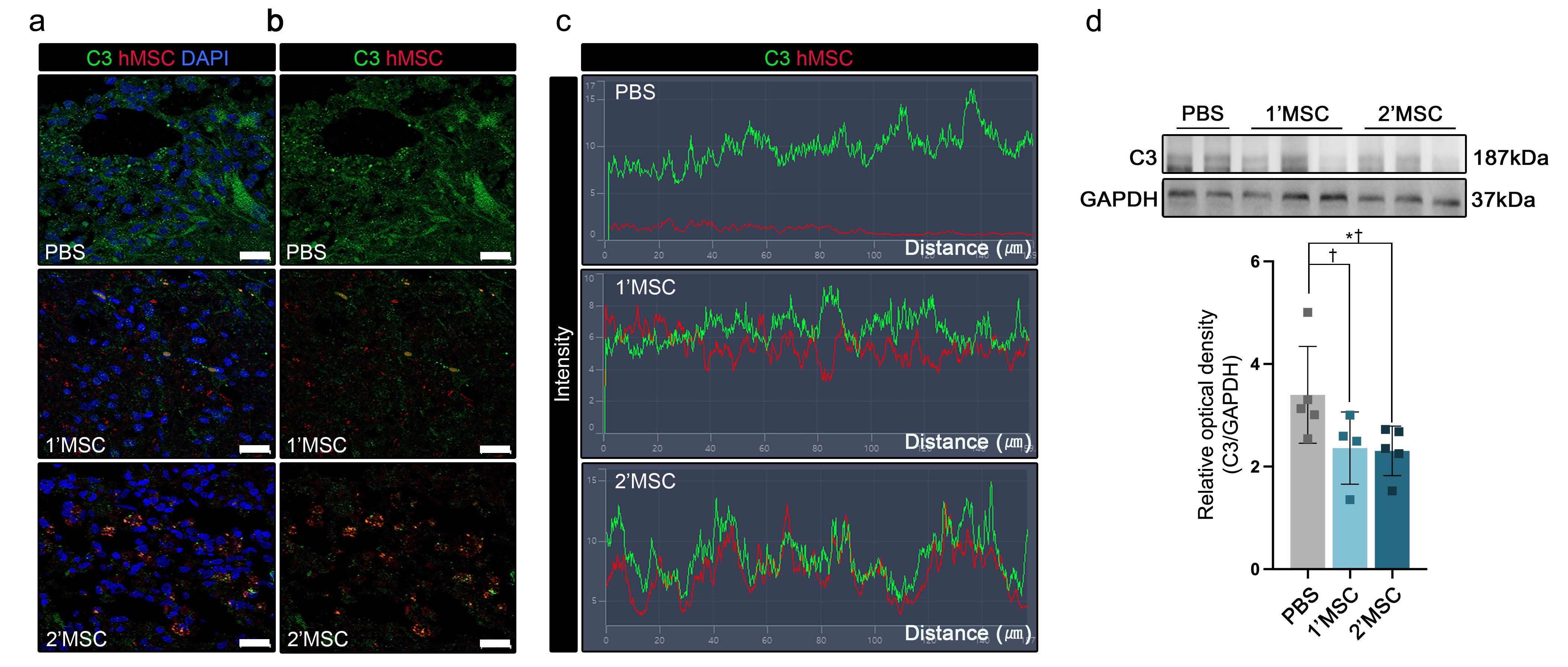

Supplement: Supplementary file 4 — Supplementary Material 3 [file 13287_2024_3714_MOESM4_ESM.jpg]

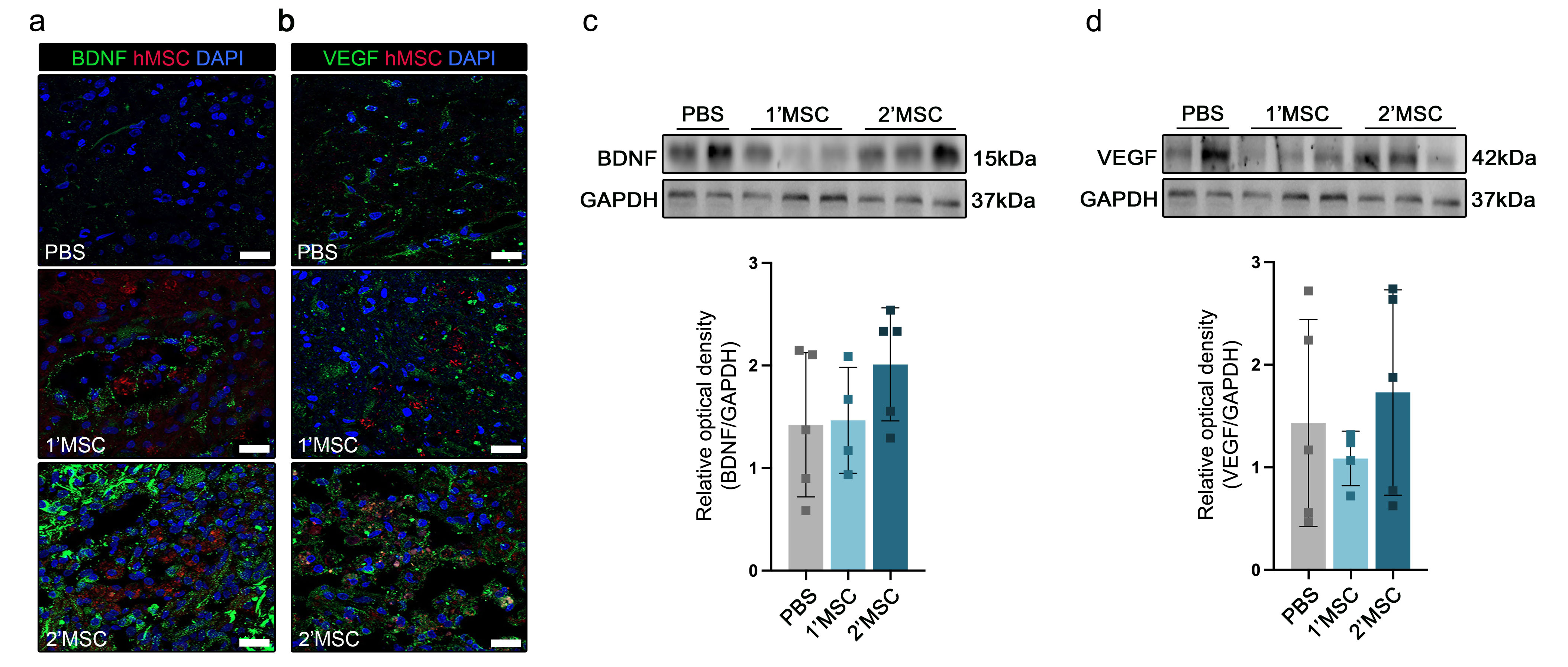

Supplement: Supplementary file 5 — Supplementary Material 4 [file 13287_2024_3714_MOESM5_ESM.jpg]

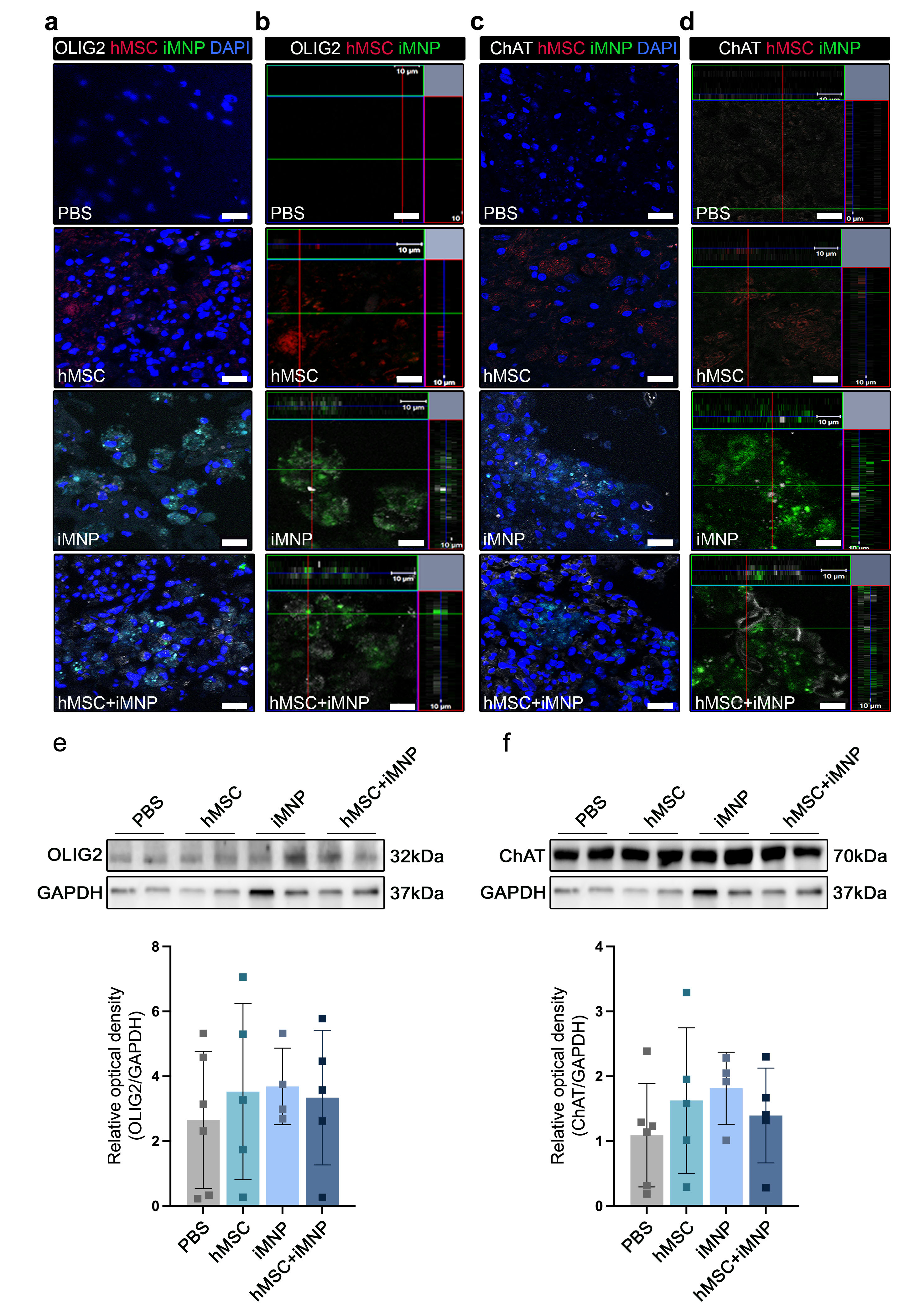

Supplement: Supplementary file 6 — Supplementary Material 5 [file 13287_2024_3714_MOESM6_ESM.jpg]

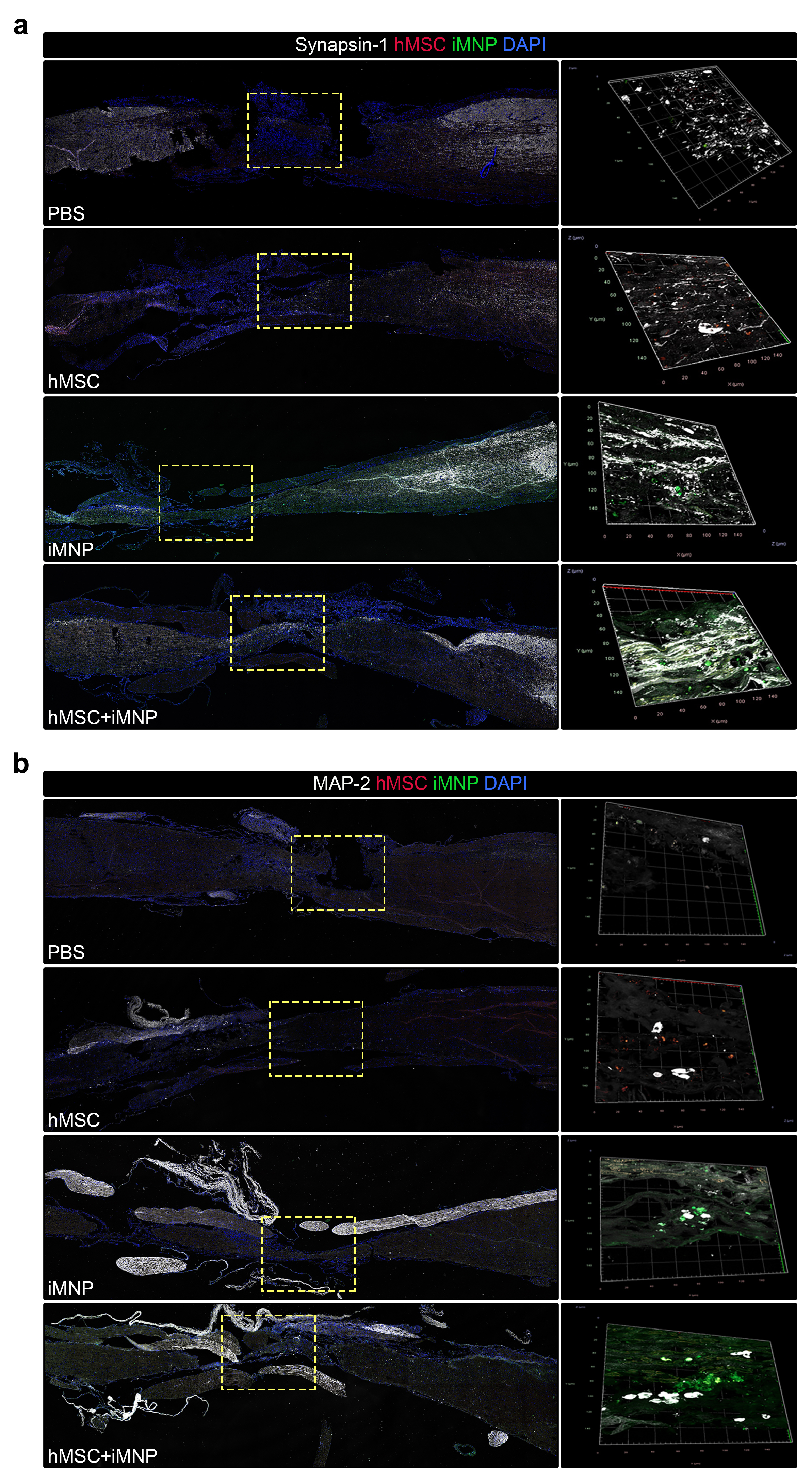

Supplement: Supplementary file 7 — Supplementary Material 6 [file 13287_2024_3714_MOESM7_ESM.jpg]
