# Supplementary material for: Stepwise combined cell transplantation using mesenchymal stem cells and induced pluripotent stem cell-derived motor neuron progenitor cells in spinal cord injury
Source: Stem Cell Res Ther. 2024 Apr 23;15:114. doi: 10.1186/s13287-024-03714-3 (PMC11036722; doi:10.1186/s13287-024-03714-3)
Supplement: Supplementary file 8 — Supplementary Material 8 [file 13287_2024_3714_MOESM8_ESM.pdf]

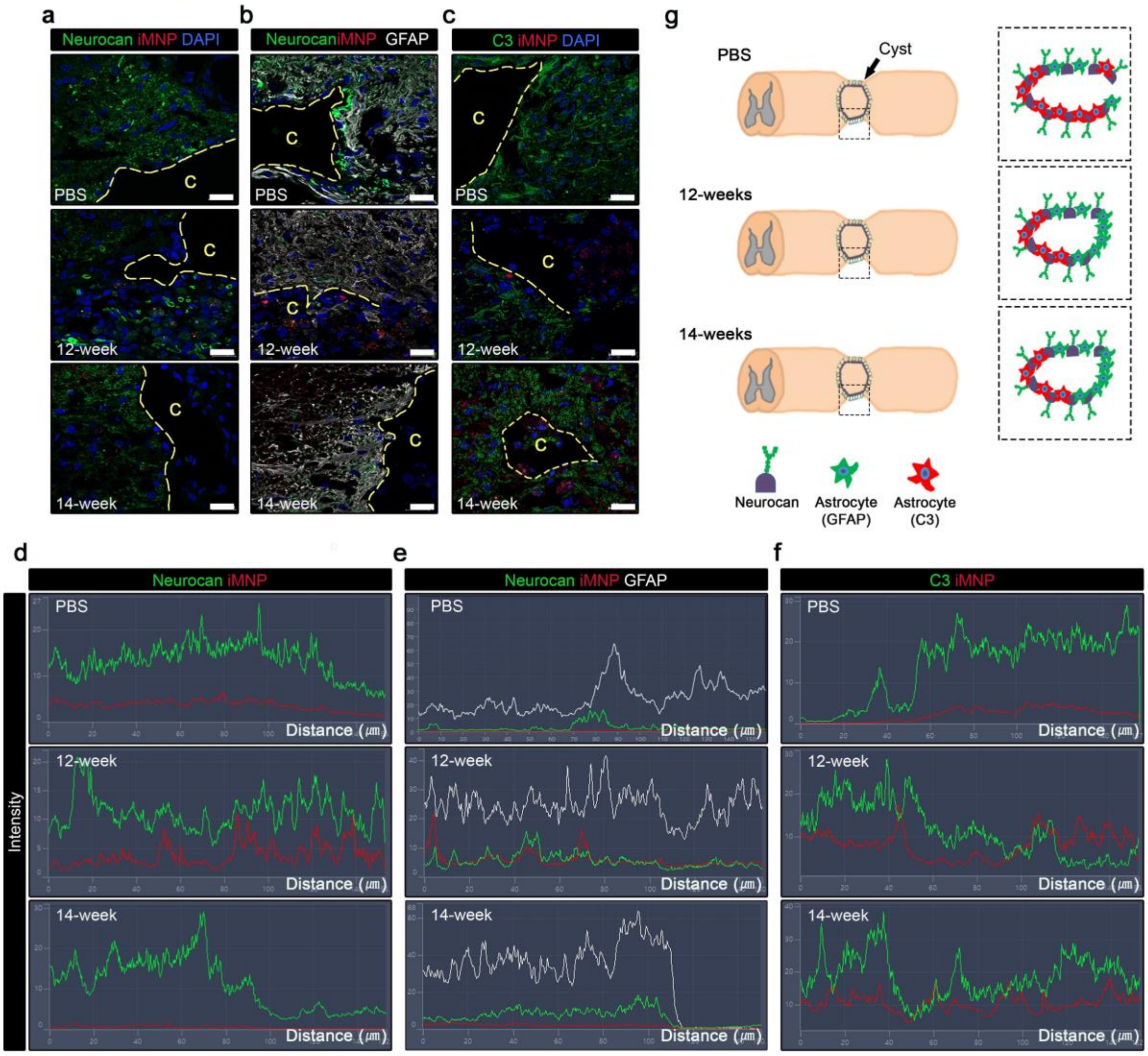

Additional file 1: Figure S1. Traumatic gliosis scar formation occurs in chronic SCI lesions. a Injured spinal cord section from a chronic SCI rat model at 12 weeks post SCI. IF staining shows the expression of neurocan in the cystic area. **b** Multi-fluorescent, confocal images show the expression of neurocan (green) and GFAP (white) in the cystic area of the posterior white matter. **c** IF staining show the expression of C3 in the cystic area. **d-f** The intensity profiles of the neurocan, GFAP, C3 (green), and iMNP (red) confocal image signals in the cystic area. **g** Images of the expression of neurocan, GFAP, and C3 in the cystic area in a chronic SCI model. The yellow dotted line is the cystic area (C). Scale bars = 20 µm. SCI, spinal cord injury; IF, immunofluorescence; GFAP, glial fibrillary acidic protein; iMNP, induced pluripotent stem cell-derived motor neuron progenitor cells.

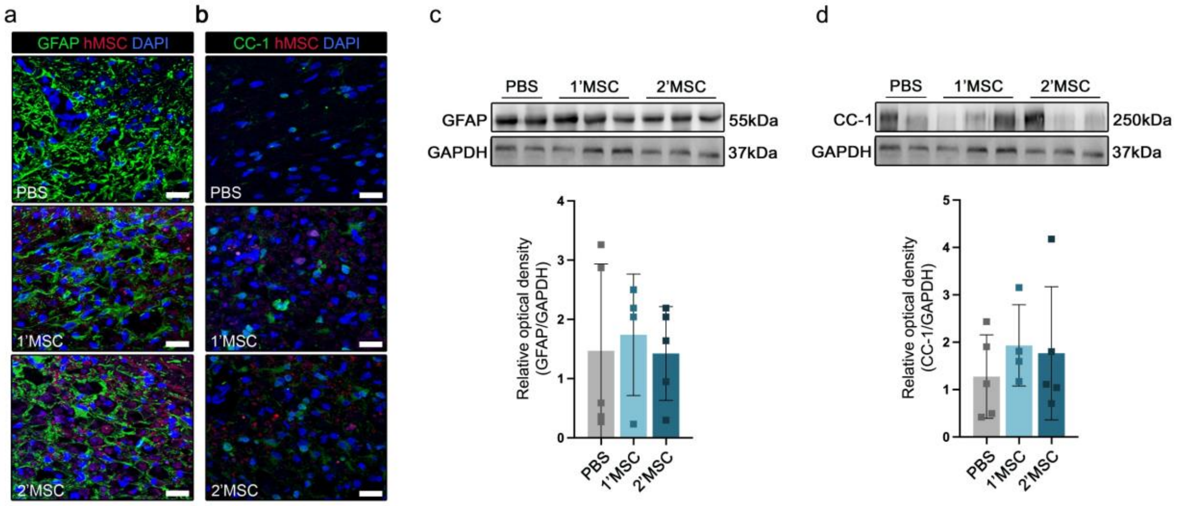

Additional file 2: Figure S2. Astrocyte and oligodendrocyte differentiation of the transplanted hMSC at acute SCI.

a Confocal images show the expression of GFAP (green) and hMSC in the white matter. **b** Confocal images show expression of CC-1 (green) and hMSC in white matter. **c**WB results of glial fibrillary acidic protein expression at the lesion site segments. **d** WB results of CC-1 expression at the lesion site segments. Full-length western blot images are presented in Additional file 7: Fig.7. (WB analysis: PBS n = 5, 1’MSC n = 4 and 2’MSC n = 5). GFAP, glial fibrillary acidic protein; WB, Western blot.

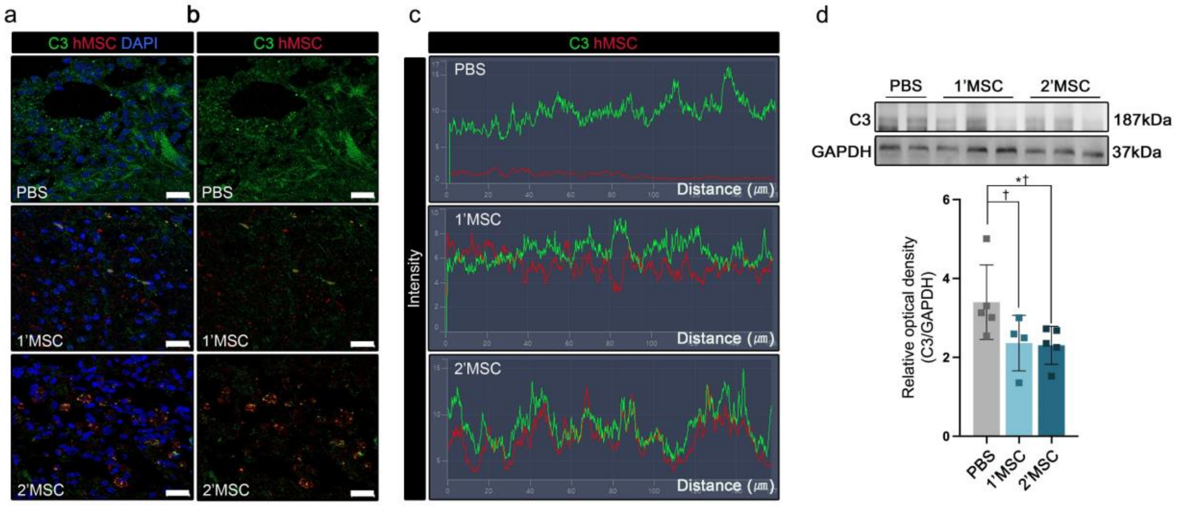

Additional file 3: Figure S3. Effects of reducing C3 expression through hMSC injection in the lesion site. a IF staining shows the expression of C3 (green) in the cystic area. **b** Multi-fluorescent, confocal images show the expression of C3 and hMSC (red) in the cystic area. The intensity profiles of the C3 and hMSC expression confocal image signals in the cystic area. **c** WB results of C3 expression in the lesion site segments (~1 cm). WB results of C3 expression in the 1'MSC and 2'MSC groups were significantly lower than those in the PBS group. Full-length western blot images are presented in Additional file 7: Fig.7.The data are presented as mean ± SEM. Statistical significance was estimated using the Kruskal–Wallis test with post hoc analysis and the Mann–Whitney (†) test with least significant difference post hoc analysis (*); *, † $P < 0.05$. (WB analysis: PBS n = 5, 1'MSC n = 4 and 2'MSC n = 5). (1' MSC = single injection, at 24 h post injury, 2'MSC = multiple injection). Scale bars = 20 μm. hMSC, human mesenchymal stem cells; IF, immunofluorescence; WB, Western blot.

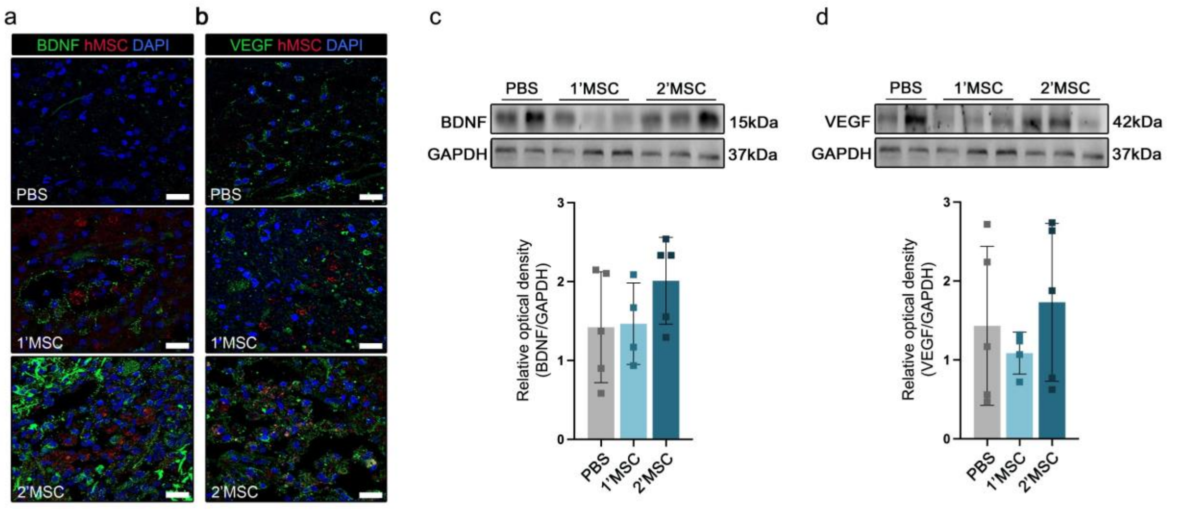

Additional file 4: Figure S4. Effects of increasing growth factors expression through

multiple hMSC injections in the lesion site. a IF staining shows the expression of BDNF (green) in the cystic area. Multi-fluorescent, confocal images show the expression of BDNF and hMSC (red) in the cystic area. **b** IF staining shows the expression of VEGF (green) in the cystic area. **c** WB results of the BDNF expression in the lesion site segments (~1 cm). WB results show that BDNF expression was higher in the 2'MSC group than those in the PBS and 1'MSC groups. **d** WB results show that VEGF expression was higher in the 2'MSC group than those in the PBS and 1'MSC groups. However, significant difference was not observed among all groups. Full-length western blot images are presented in Additional file 7: Fig.7. The data are presented as mean ± SEM. Statistical significance was estimated using the Kruskal–Wallis test with post hoc analysis and the Mann–Whitney (†) test with least significant difference post hoc analysis (*); *, † $P < 0.05$. (WB analysis: PBS n = 5, 1'MSC n = 4 and 2'MSC n = 5). (1' MSC = single injection, at 24 h post injury, 2'MSC = multiple injection). Scale bars = 20 μm. hMSC, human mesenchymal stem cells; IF, immunofluorescence; WB, Western blot; BDNF, brain-derived neurotrophic factor; VEGF, vascular endothelial growth factor.

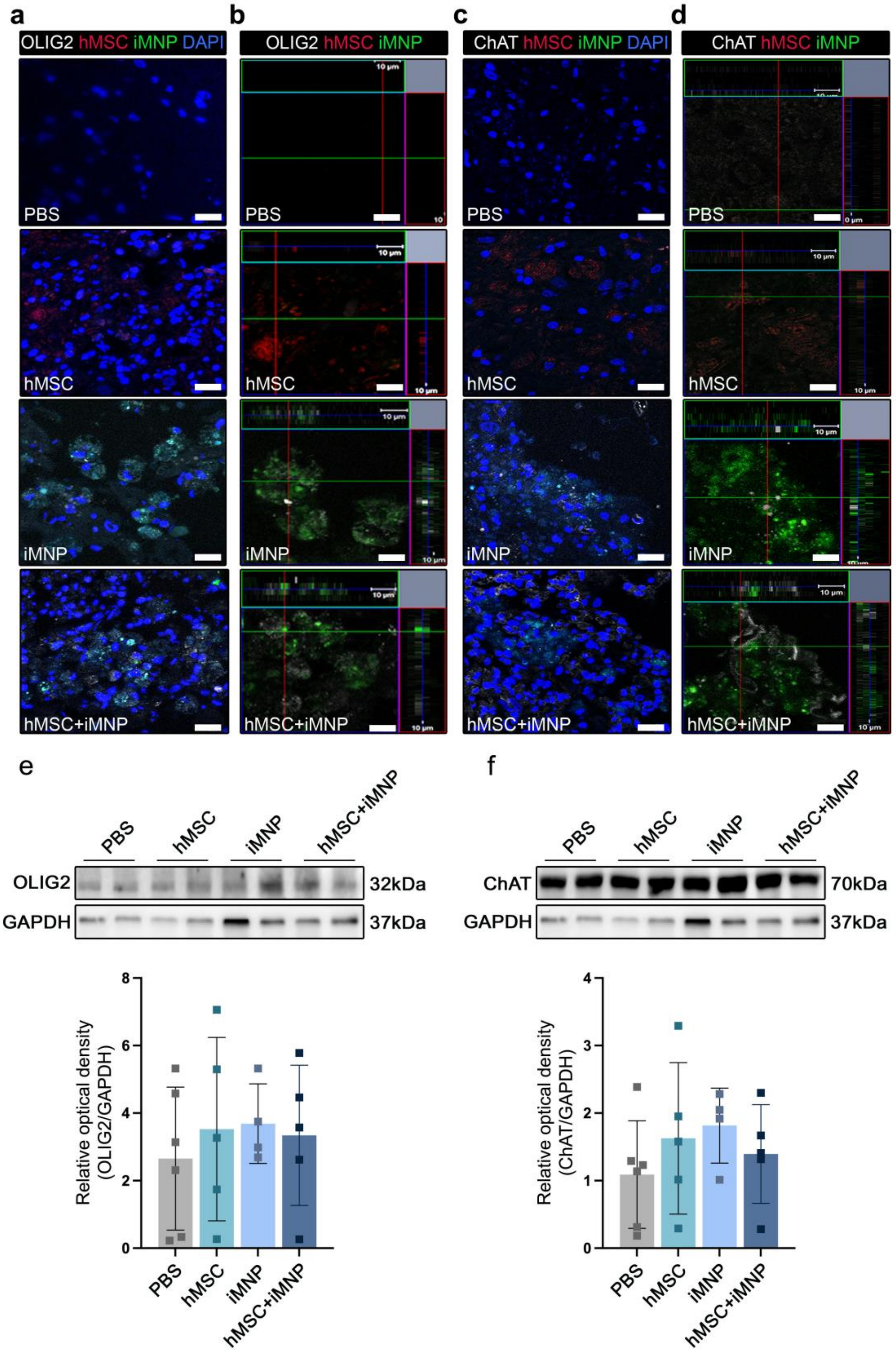

Additional file 5: Figure S5. Enhancements of motor neuron differentiation through iMNP transplantation for an SCI model. a IF staining shows the expression of OLIG2 (white) in the cystic area. Multi-fluorescent, confocal images show the expression of OLIG2

with hMSC (red) and iMNP (green) in the cystic area. **b** Orthogonal view of the confocal Z-stack image of OLIG2 with hMSC and iMNP expression. **c** IF staining shows the expression of ChAT (white) in the gray matter of the cystic area. **d** Orthogonal view of the confocal Z-stack image of ChAT with hMSC and iMNP expression. **e** WB results of OLIG2 expression in the lesion site segments (~1cm). **f** WB results of ChAT expression in the lesion site segments (~1cm). Full-length western blot images are presented in Additional file 7: Fig.7. The data are presented as mean ± SEM. Statistical significance was estimated using the Kruskal–Wallis test with post hoc analysis and the Mann–Whitney (†) test with least significant difference post hoc analysis (*); *, † $P < 0.05$. (WB analysis: PBS n = 6, hMSC n = 5, iMNP n = 4 and hMSC + iMNP n = 5,) Scale bars = 20 and 10 μm. iMNP, induced pluripotent stem cell-derived motor neuron progenitor cells; SCI, spinal cord injury; IF, immunofluorescence; hMSC, human mesenchymal stem cells; ChAT, choline acetyltransferase; WB, Western blot.

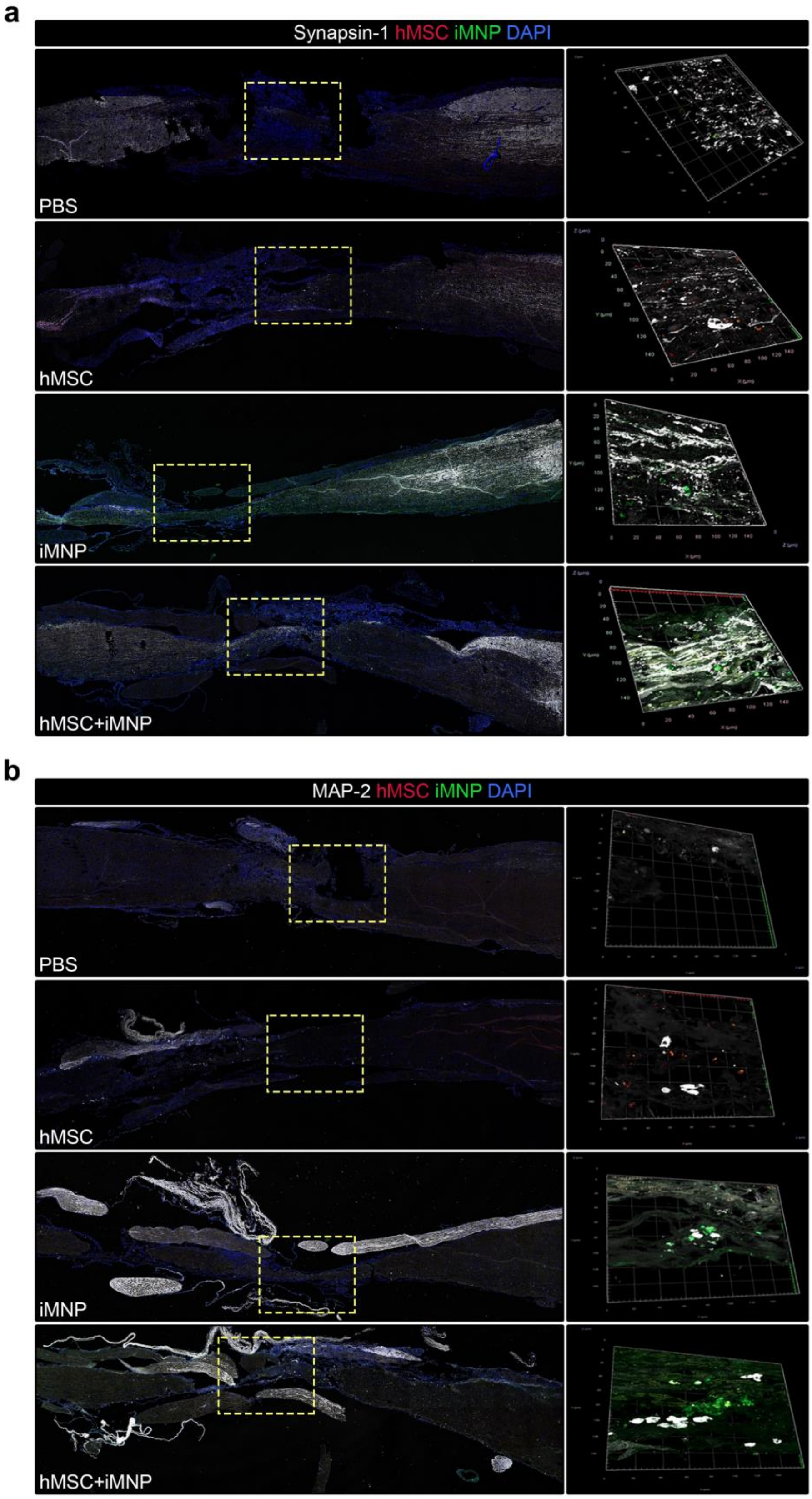

Additional file 6: Figure S6. Expression of Synapin-1 and MAP-2 expression in the

lesion site.

a IF staining images show tiled mosaic scans of longitudinal sections from representative lesion site segments (~1cm). IF staining of Synapsin-1 shows tiled mosaic scans of longitudinal section from lesion site segment. Right confocal images of longitudinal section indicate the magnified region of interest using 3D images. **b** IF staining of MAP-2 shows tiled mosaic scans of longitudinal section from lesion site segment. Right confocal images of longitudinal section indicate the magnified region of interest using 3D images.
